# Hypoxia-induced USP13 expression drives ferroptosis resistance and tumor immune evasion in hepatocellular carcinoma through the stabilization of ACLY

**DOI:** 10.1038/s41420-025-02869-z

**Published:** 2025-12-02

**Authors:** Kuan Hu, Juanni Li, Kui Chen, Xingyu Mi, Yilin Pan, Jianing Tang, Jing Cao, Xiao Zhong

**Affiliations:** 1 Department of Liver Surgery, Xiangya Hospital, Central South University, Changsha, Hunan China; 2 Department of General Surgery, Xiangya Hospital, Central South University, Changsha, Hunan China; 3 Department of Pathology, Xiangya Hospital, Central South University, Changsha, Hunan China; 4 Department of Infectious Diseases, Xiangya Hospital, Central South University, Changsha, Hunan China; 5 Hunan Key Laboratory of Viral Hepatitis, Xiangya Hospital, Central South University, Changsha, Hunan China

**Keywords:** Hepatocellular carcinoma, Cancer metabolism

## Abstract

Hepatocellular carcinoma (HCC) is an aggressive liver cancer with high recurrence and poor prognosis. This study aims to explore USP13’s role in HCC progression and assess its potential as a therapeutic target to induce ferroptosis and enhance immune response. HCC patient-derived organoids (PDOs), HCC cell lines and animal models were utilized to evaluate the anti-cancer responses of USP13 inhibition. We analyzed the correlation of USP13 expression and immune cell infiltration using single-cell RNA sequencing, flow cytometry analysis. A USP13 inhibitor, 2-Methoxyestradiol (2-Met), was used to evaluate its therapeutic efficacy. USP13 was found to be highly expressed in HCC tissues and was correlated with poor prognosis. Single-cell RNA sequencing analysis indicated that high expression of USP13 in HCC cells was associated with decreased enrichment of CD8 + T cells in the tumor microenvironment (TME). Targeting USP13 reduced HCC cell proliferation, stemness, and cholesterol metabolism while promoting ferroptosis and enhancing T cell-mediated cytotoxicity. Mechanistically, USP13 stabilized ACLY via inhibiting the K48-specific poly-ubiquitination process on ACLY protein at the K726 site. Under hypoxia condition, HIF-1α upregulates the transcription of USP13 by binding to its promoter region, which stabilizes ACLY protein. Overall, this research reveals that hypoxia-induced USP13 expression drives ferroptosis resistance and tumor immune evasion in hepatocellular carcinoma through the stabilization of ACLY. Pharmacological inhibition or knockdown of USP13 impedes HCC progression, induces ferroptosis, and enhances T cell-mediated cytotoxic effects. These results highlight that USP13 could be a promising therapeutic target for HCC.

## Introduction

Hepatocellular carcinoma (HCC) is the most common form of primary liver cancer, accounting for approximately 90% of all cases worldwide [1, 2]. HCC is characterized by aggressive tumor growth, rapid progression, and high recurrence rates, leading to poor clinical outcomes [3, 4]. Despite advancements in treatment modalities such as surgical resection, chemotherapy, radiotherapy, targeted therapy, and immunotherapy, the overall prognosis for HCC remains dismal, particularly in advanced stages where therapeutic options are limited [5, 6]. The five-year survival rate for patients with HCC is less than 15%, even after comprehensive treatment strategies [7]. Consequently, there is an urgent need to develop more effective therapeutic approaches to improve outcomes for HCC patients.

Ubiquitination is a post-translational modification that regulates numerous cellular processes, including cell cycle progression, apoptosis, DNA repair, and protein degradation. This dynamic process is mediated by a cascade of three enzymes: the ubiquitin-activating enzyme (E1), the ubiquitin-conjugating enzyme (E2), and the ubiquitin ligase (E3) [8, 9]. Dysregulation of ubiquitination has been implicated in various human cancers, where aberrant ubiquitin signaling can lead to uncontrolled cell proliferation and tumorigenesis [10, 11]. For instance, the overexpression of the E3 ligase SKP2, which promotes the degradation of the cyclin-dependent kinase inhibitor p27, facilitating tumor progression in lung and prostate cancers [12, 13]. The E3 ligase TRIM25 has been shown to drive cell proliferation and migration by forming a complex with p53 and MDM2 in lung cancer [14]. In contrast to the ubiquitination process, deubiquitinating enzymes (DUBs) remove ubiquitin from target proteins, thereby reversing ubiquitin signaling and providing a regulatory mechanism [15].

DUBs are a family of enzymes that control protein stability and function by cleaving ubiquitin chains. There are approximately 100 DUBs encoded by the human genome, and they are classified into six families based on their sequence and catalytic domains [16, 17]. Many DUBs have been implicated in tumorigenesis, acting as either oncogenes or tumor suppressors depending on their specific substrates [18]. For instance, USP7 (ubiquitin-specific protease 7) promotes non-small-cell lung cancer (NSCLC) cell metabolism by activating c-Abl and HK2 [19]. Similarly, USP28 stabilizes MYC, a potent oncogene, thereby promoting tumor growth in breast cancer [20]. USP1 has been shown to regulate key oncogenic pathways, making it attractive therapeutic target in cancer [21]. In recent years, DUB inhibitors have emerged as a promising class of cancer therapeutics, with several studies demonstrating the potential of these inhibitors to suppress tumor growth by modulating DUB activity [22, 23], while their potential anti-tumor effects in HCC have been relatively underexplored.

Among the DUBs, ubiquitin-specific protease 13 (USP13) has attracted attention due to its role in regulating cellular processes relevant to cancer progression [24, 25]. 2-Methoxyestradiol (2-Met), an inhibitor of USP13 from a natural compound library, has been shown to exhibit promising anti-tumor effects in some cancers [26, 27]. Research has shown that 2-Met suppresses USP13 activity, particularly in NSCLC, where it inhibits the deubiquitination of key oncogenic proteins like β-catenin, reducing metastasis [28]. In ovarian cancer, 2-Met has demonstrated efficacy in promoting cancer cell death after replication stress by inhibiting USP13 [29]. Additionally, studies suggest that 2-Met may play a role in suppressing the proliferation of prostate and breast cancers, highlighting its broad therapeutic potential [30, 31]. However, its specific role in HCC remains unclear and warrants further investigation.

In this study, we investigated the role of USP13 in HCC progression and explored the therapeutic potential of 2-Met as a USP13 inhibitor. Our results demonstrated that USP13 is upregulated in HCC tissues and is associated with poor patient prognosis. Furthermore, we showed that targeting USP13 effectively inhibited cell proliferation, stemness, and cholesterol metabolism in HCC cells, while promoting ferroptosis and enhancing the cytotoxic effects of T cells. Mechanistically, USP13 was found to stabilize ACLY by regulating its ubiquitination, thereby influencing key metabolic and immune pathways in HCC. Furthermore, the expression of USP13 was regulated by HIF-1α through transcriptional activation. These findings suggest that targeting USP13 may provide a novel therapeutic strategy for HCC, particularly in cases where traditional treatments have failed.

## Results

### Targeting USP13 inhibits hepatocellular carcinoma progression

**Fig. 1:**
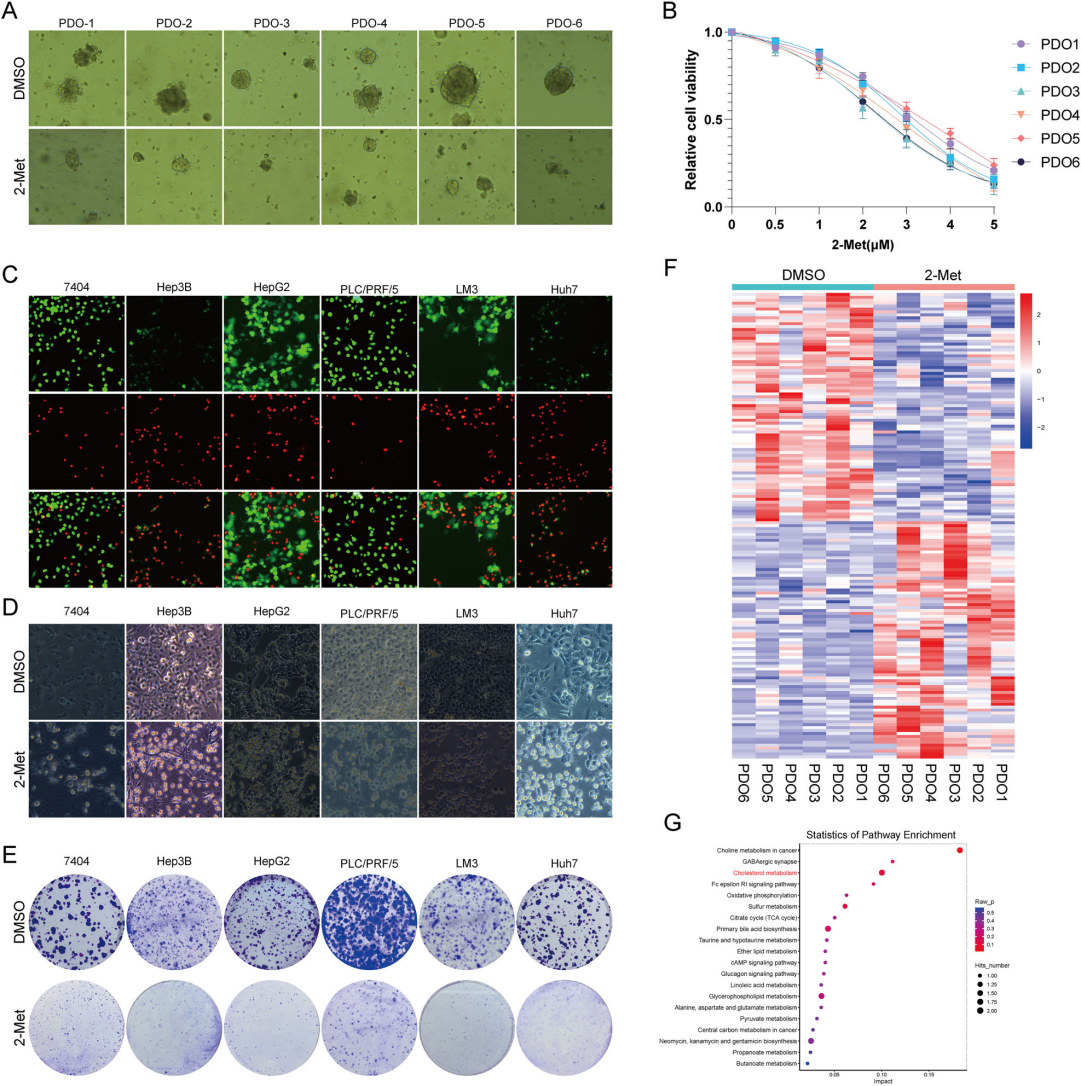
2-Methoxyestradiol (2-Met) exhibits high potency against hepatocellular carcinoma progression. **A** Representative images of PDOs treated with 2-Met (5 μm, 48 h). **B** Dose-response analysis of 6 PDOs treated with escalating concentrations of 2-Met for 48 h was conducted, with cell viability determined using the CellTiter-Glo assay. **C** HCC cells were stained with Calcein/PI following 2-Met treatment (2 μM, 48 h). Calcein-AM for live cells and PI for dead cells. **D** Representative images of the morphology of 6 HCC cells under microscopy after treatment with 2-Met (2 μM, 48 h). **E** HCC cells were stained with crystal violet following 2-Met treatment (2 μM, 48 h). A total of 5 × 10⁴ cells were seeded in 6-well plates, and 3 days later, treated with DMSO or 2-Met for 48 h. Cells were then washed and stained using crystal violet. **F** Heatmap analysis showed substantial changes in metabolic patterns of HCC PDOs following 2-Met treatment (1 μM, 48 h). **G** Pathway enrichment analysis showed numerous metabolic pathways were impacted in 2-Met group, with cholesterol metabolism being notably affected. These results presented are representative of three independent experiments.

We explored the anti-tumor effect of 2-Met, an inhibitor of USP13 from a natural compound library. To this end, six patient-derived organoids (PDOs) were generated from primary liver cancer tissues, and the effects of 2-Met on cell viability were assessed. Our results demonstrated that 2-Met exerted significant anti-tumor activity in the PDO models (Fig. 1A, B). Besides, Calcein AM/PI staining assays indicated that treatment with 2-Met led to an elevation in cell death (Fig. 1C). Consistently, we observed a reduction in the number of surviving cells in the 2-Met treatment group compared to the control, as demonstrated by cell morphology under microscopy (Fig. 1D). Additionally, crystal violet staining confirmed that the 2-Met treatment resulted in increased cell death (Fig. 1E). Furthermore, we found that USP13 expression was significantly higher in HCC tissues compared to normal liver tissues (Supplementary Fig. S1A). Survival analysis further indicated that elevated USP13 levels were associated with poor prognosis (Supplementary Fig. S1B). Collectively, these findings suggest that USP13 may be a valuable prognostic marker.

To elucidate the mechanisms behind the aforementioned findings, we conducted metabolomics analysis of HCC PDOs using lipid chromatography-mass spectrometry (LC-MS)-based metabolomics analysis. Heatmap analysis showed substantial changes in metabolic patterns following 2-Met treatment (Fig. 1F). Pathway enrichment analysis of the significantly altered metabolites indicated that numerous metabolic pathways were impacted in the 2-Met treatment group, with cholesterol metabolism being notably affected (Fig. 1G).

### USP13 drives cell proliferation, stemness, and cholesterol metabolism while inhibiting ferroptosis

**Fig. 2:**
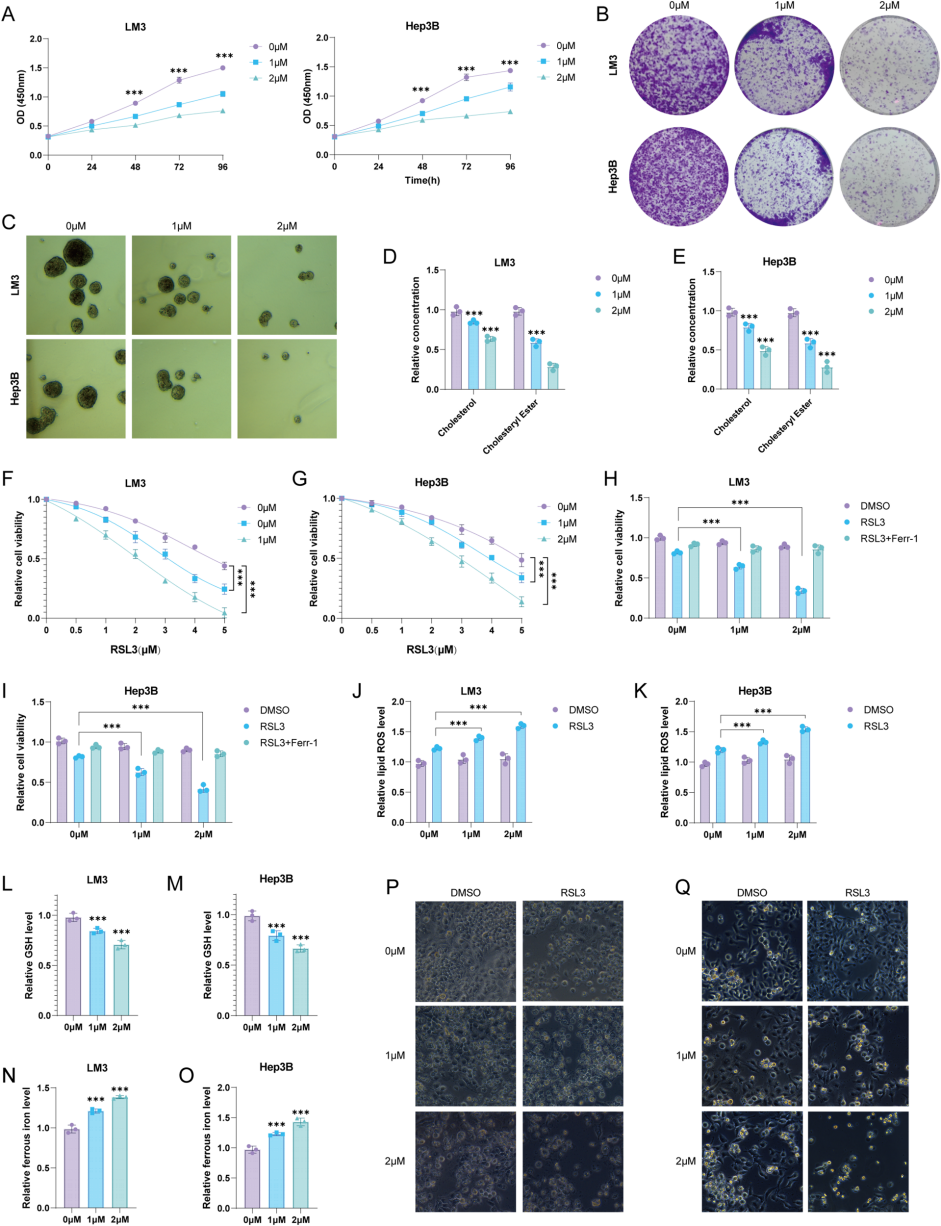
2-Met inhibits cell proliferation, stemness, and cholesterol metabolism while promoting ferroptosis. **A** CCK8 assays were performed on cells. **B** Colony formation assays were performed on cells. LM3 and Hep3B cells were plated in 6-well plates at a density of 2000 cells per well and treated with the specified concentration of 2-Met for 2 weeks. The cells were subsequently rinsed with PBS and stained using 0.5% crystal violet. **C** Tumorsphere formation assays were performed on cells. Cells are plated at a density of 2 × 10^3^ cells per well in ultra-low attachment 6-well plates treated with the specified concentration of 2-Met for 2 weeks, and after two weeks, spheres are counted using a microscope. **D**, **E** The concentration of cholesterol and cholesteryl ester in LM3 and Hep3B cells treated with the specified concentration of 2-Met were evaluated. **F**, **G** The LM3 and Hep3B cells viability were assessed under a set 2-Met concentration as the RSL3 concentration was progressively increased (48 h). **H**, **I** the CCK8 assay was used to assess the response of 2-Met-treated LM3 and Hep3B cells to RSL3 (5 μM) with or without ferrostatin (1 μM). HCC cells were treated with indicated concentration of 2-Met for 24 h, then the response of 2-met -treated HCC cells to RSL3 (5 μM)±ferrostatin (1 μM) was detected using CCK8 assay. **J**, **K** Lipid ROS levels were measured in LM3 and Hep3B cells following 24-h treatment with the indicated concentration of 2-Met, with and without RSL3. **L**, **M** GSH levels were measured in LM3 and Hep3B cells following 24-h treatment with the indicated concentration of 2-Met. **N**, **O** Ferrous iron levels were measured in LM3 and Hep3B cells following 24-h treatment with the indicated concentration of 2-Met. **P**, **Q** Representative images of cell morphology were photographed in LM3 and Hep3B cells. HCC cells were treated with RSL3 (5 μM) and indicated concentration of 2-Met for 24 h. Data are expressed as mean ± SD based on triplicate biological samples. Statistical significance is defined as *p < 0.05, **p < 0.01, and ***p < 0.001.

We proceeded to assess the anti-tumor efficacy of 2-Met. Results from the CCK8 assay indicated that 2-Met markedly decreased the proliferation of LM3 and Hep3B cells (Fig. 2A). Similarly, the colony formation assay demonstrated a significant reduction in the clonogenic capacity of HCC cells upon 2-Met treatment (Fig. 2B). Next, we investigated the impact of 2-Met on the stemness properties of HCC cells. Our findings showed that 2-Met markedly decreased oncosphere generation in both HCC cells (Fig. 2C). Additionally, prior metabolomics studies indicated a close association between 2-Met and cholesterol metabolism. In line with this, our findings demonstrated that 2-Met markedly decreased cholesterol and cholesteryl ester levels in both HCC cells (Fig. 2D, E). Several studies have suggested a link between cholesterol metabolism and ferroptosis. We next examined the potential association between 2-Met and ferroptosis in our study. We found that 2-Met reduced cell viability following RSL3 treatment (Fig. 2F, G). We further investigated the involvement of 2-Met in ferroptosis by assessing RSL3-induced ferroptosis with or without ferrostatin-1 (Ferr-1), a known ferroptosis inhibitor. The findings revealed that Ferr-1 was able to reverse the reduction in cell viability caused by 2-Met under RSL3 treatment, suggesting that the effect is specifically linked to ferroptosis (Fig. 2H, I). Upon RSL3 treatment, we found that 2-Met elevated lipid ROS levels (Fig. 2J, K). Moreover, GSH, a critical metabolite in glutathione metabolism, was significantly reduced in 2-Met-treated cells (Fig. 2L, M). We also noted an upregulation of ferrous iron levels with 2-Met treatment (Fig. 2N, O). Microscopic analysis further confirmed that 2-Met reduced cell viability in the presence of RSL3 (Fig. 2P, Q).

As 2-Met is identified as a USP13 inhibitor. We directly knocked down USP13 in HCC cells. Consistently, USP13 knockdown suppressed cell proliferation and stemness, along with a reduction in cholesterol and cholesteryl ester expression levels (Fig. S2A–E). Additionally, USP13 knockdown inhibited cell viability under RSL3 treatment, an effect that could be rescued by Ferrostatin-1 (Ferr-1), suggesting that this mechanism is specifically involved in cell ferroptosis (Fig. S2F–I). Moreover, silencing USP13 enhanced lipid ROS levels under RSL3 treatment, decreased GSH levels, and increased ferrous iron concentrations (Fig. S2J–O). Microscopic examination of cell morphology confirmed that USP13 knockdown significantly impaired cell viability under RSL3 treatment (Fig. S2P, Q).

### USP13 mediates T cell function in hepatocellular carcinoma

**Fig. 3:**
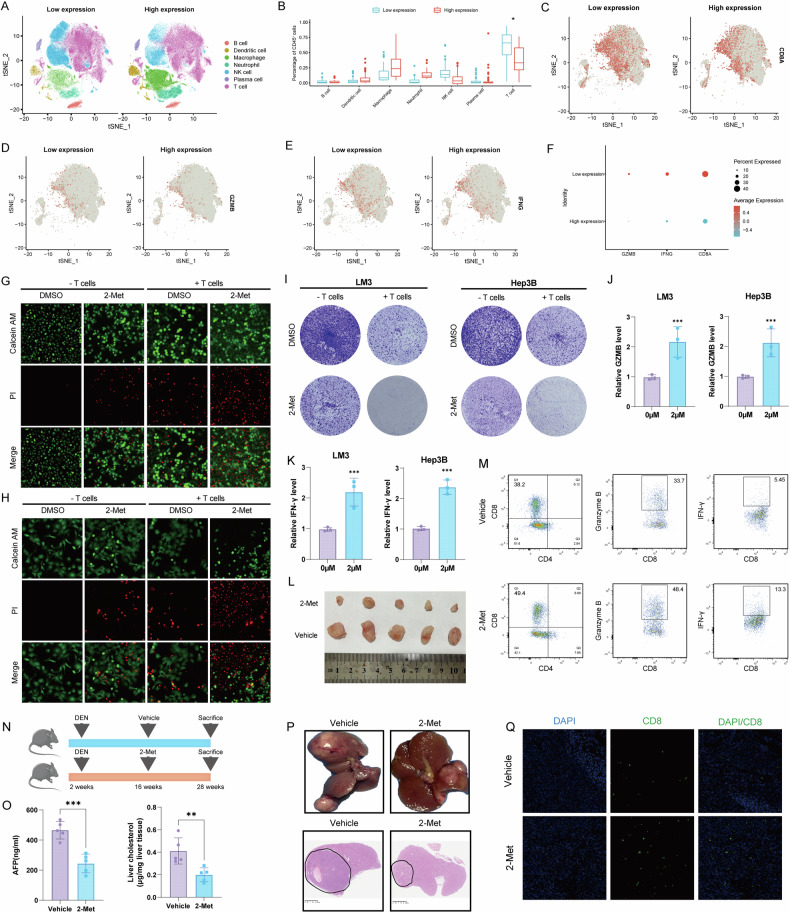
2-Met enhances T cell function and inhibits the progression of primary hepatocellular carcinoma in mice. **A**, **B** The t-SNE plot and box plot of major immune cell types in USP13-low expression and USP13-high expression HCC tumors. **C**–**F** Density t-SNE plot and dot plot for the expression of CD8, GZMB and IFNG in infiltrated T-cells within USP13-low expression and USP13-high expression HCC tumors. **G**, **H** Calcein/PI staining indicated 2-Met treatment increased T cell-mediated tumor cell death. HCC cells pretreated with or without 2-Met (2 μM) were co-cultured with T cell, 72 h after co-incubation, tumor cells were collected and stained with Calcein-AM/PI. **I** Crystal violet staining assays indicated that 2-Met treatment increased T cell-mediated tumor cell death. HCC cells pretreated with or without 2-Met (2 μM) were co-cultured with T cell, 72 h after co-incubation, tumor cells were collected and stained with crystal violet. **J**, **K** HCC cells pretreated with or without 2-Met (2 μM) were co-cultured with T cell, 72 h after co-incubation, GZMB and IFN-γ levels were measured in supernatant determined by ELISA. **L** C57BL/6 Xenograft data show that 2-Met markedly inhibits HCC tumor growth in vivo. **M** Flow cytometric analysis of infiltrating CD8 + T cells in Hepa1-6 tumors. **N** The schematic diagram illustrating the administration of 2-Met to DEN-induced primary hepatocellular carcinoma mice. **O** AFP and liver cholesterol levels were measured in primary hepatocellular carcinoma mice. **P** Representative images of livers and HE-stained microscopic images are presented. **Q** Representative fluorescence microscopy images were used to assess the infiltration of CD8 + T cells in DEN-induced primary hepatocellular carcinoma mice treated with vehicle or 2-Met. Data are expressed as mean ± SD based on triplicate biological samples. Statistical significance is defined as *p < 0.05, **p < 0.01, and ***p < 0.001.

Cholesterol has been reported to induce CD8 + T cell exhaustion in the tumor microenvironment [32]. In this research, we found that 2-Met significantly influenced the cholesterol metabolism pathway in treated cells. Therefore, we examined whether targeting USP13 influences CD8 + T cell infiltration in HCC. We analyzed publicly available scRNA-seq datasets (GSE125449, GSE149614, GSE151530, GSE156625, GSE162616, GSE166635, GSE189903, GSE202642, GSE210679, GSE242889, GSE282701) and divided samples into USP13-Low expression or USP13-High expression group according to the USP13 expression in tumor cells. We observed that a significant reduction of T cells in USP13-High expression group (Fig. 3A, B). Compared with those of the USP13-Low expression group, the infiltrated T-cells of USP13-High expression tumors had lower expression of CD8, Granzyme B and IFN-γ expression (Fig. 3C–F). These results suggest that USP3 may facilitate the suppressive TME within HCC tumors. We further investigated whether targeting USP13 modulates CD8 + T cell functions in HCC. T cells were isolated from human peripheral blood and co-cultured with HCC cells, either with or without 2-Met treatment. As shown in Fig. 3G, H revealed by Calcein AM/PI staining assays, more cell death was observed in the co-culture group with T cells. Additionally, HCC cells treated with 2-Met were more sensitive to T cell-mediated tumor cell death, resulting in increased cell death caused by T cells (Fig. 3G, H). Consistently, cell death was significantly increased in the T cell co-culture group, and the addition of 2-Met further exacerbated cell death in this group, as demonstrated by crystal violet staining (Fig. 3I). As we known, once CD8 + T cells are activated, they secrete Granzyme B and IFN-γ, two key molecules that work together to enable these cells to combat infections, manage tumor growth, and fine-tune immune regulation. Next, we evaluated secreted GZMB and IFN-γ levels in supernatant by ELISA. Our results demonstrated that HCC cells treated with 2-Met led to a marked upregulation of both GZMB and IFN-γ secreted by CD8 + T cells (Fig. 3J, K). Additionally, using C57BL/6 xenograft models, we explored the role of 2-Met in HCC in vivo, with the data indicating that 2-Met treatment substantially reduced tumor growth in vivo (Fig. 3L). Consistent results were obtained through flow cytometry analysis, which showed a notable increase in the population of infiltrating CD8 + T cells in the tumors and elevated levels of GZMB+ and IFN-γ+ in CD8 + T cells in the 2-Met treatment group (Fig. 3M). We further examined the anti-tumor function of 2-Met using *DEN*-induced primary hepatocarcinoma (Fig. 3N). AFP and liver cholesterol levels were markedly decreased in the 2-Met group (Fig. 3O). Figure 3Q display representative images of liver gross specimens and histological images of liver cancer tissues. The results show a significant reduction in tumor size in the 2-Met treatment group (Fig. 3P). Immunofluorescence analysis revealed a substantial increase in the infiltration of CD8 + T cells within the liver cancer tissues under 2-Met treatment (Fig. 3Q). These in vitro and in vivo findings indicate that 2-Met enhances the infiltration and activation of CD8 + T cells and intensifies their cytotoxic effects against tumor cells.

We also found that USP13 depletion delayed tumor growth in both immunocompetent C57BL/6 mice and immunodeficient NCG mice transplanted with Hepa 1–6 tumor cells; however, the effect was weaker in the NCG mice, underscoring the importance of intact adaptive immunity in the efficacy of USP13 depletion (Fig. S3A–C). And consistent with our previous findings from 2-Met treatment, knockdown of USP13 significantly promoted liver cancer cell death in co-culture with T cells and inhibited cell proliferation (Supplementary Fig. S3D–G). Additionally, USP13 knockdown markedly increased the secretion of GZMB and IFN-γ (Supplemental Fig. S3H, I). In vivo experiments further confirmed that silencing USP13 led to a significant reduction in tumor size (Supplementary Fig. S3J), accompanied by an increase in both CD8 + T cell infiltration and the expression levels of GZMB and IFN-γ within the tumor tissue (Supplementary Fig. S3K). Conversely, overexpression of USP13 using AAV8-USP13 in mice produced the opposite effects: tumor volume significantly increased (Supplementary Fig. S4A–C), AFP levels were elevated (Supplementary Fig. S4D), and the infiltration of CD8 + T cells was notably reduced (Supplementary Fig. S4E). Considering USP13’s involvement in tumor cholesterol regulation, we investigated whether its effects on T cell function and infiltration depend on cholesterol. In human PBMC-derived CD8 + T cells co-cultured with HCC cells, USP13 depletion in HCC cells elevated Granzyme B + CD8+ and IFNγ + CD8 + T populations while reducing PD-1+ and TIM-3+ exhausted subsets. Considering USP13’s involvement in tumor cholesterol regulation, we investigated whether its effects on T cell function and exhaustion depend on cholesterol. Pharmacological treatments confirmed cholesterol pathway dependency: Cholesterol supplementation reversed USP13 depletion-induced T cell function enhancement, while methyl-β-cyclodextrin (M-β-CD), a cholesterol-depleting agent, nearly abolished USP13’s regulatory effect on CD8 + T cells (Fig. S5). These in vitro and in vivo findings indicate that USP13 enhances the infiltration and activation of CD8 + T cells in liver cancer depends on cholesterol, thereby contributing to tumor suppression. These in vitro and in vivo findings indicate that USP13 enhances the infiltration and activation of CD8 + T cells in liver cancer, thereby contributing to tumor suppression.

### USP13 directly binds to ACLY

**Fig. 4:**
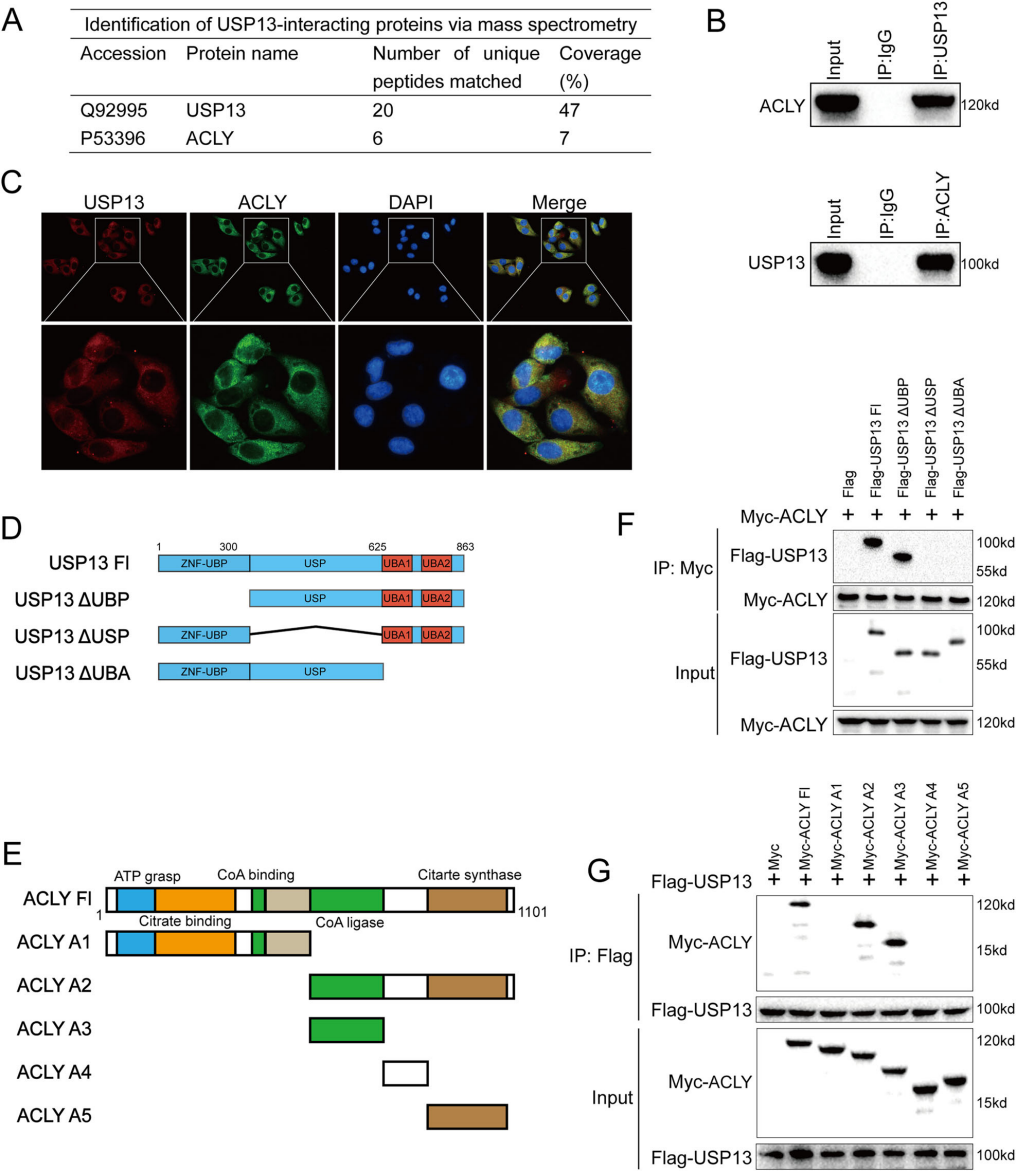
USP13 interacts with ACLY. **A** Mass spectrometry analysis of USP13-interacting proteins in LM3 cells was conducted, and the interaction between USP13 and ACLY was specifically highlighted. **B** Co-immunoprecipitation (Co-IP) assay demonstrated the interaction between endogenous USP13 and ACLY in LM3 cells. **C** Immunofluorescence analysis revealed that USP13 and ACLY exhibited partial colocalization in LM3 cells. **D**, **E** USP13 and ACLY domain structures, along with the deletion mutants utilized in this research. **F** The USP and UBA domains of USP13 were found to interact directly with ACLY. In HEK293 cells, 2 μg of Myc-ACLY was co-transfected with either full-length or mutant forms of Flag-USP13. After 24 h of incubation, cells were lysed using NP-40 buffer. A Myc antibody was utilized for co-IP, and the interaction domains of USP13 were detected via Flag antibody. **G** The CoA ligase domain of ACLY was found to interact directly with USP13. In HEK293 cells, 2 μg of Flag-USP13 was co-transfected with either full-length or mutant forms of Myc-ACLY. After 24 h of incubation, cells were lysed using NP-40 buffer. A Flag antibody was utilized for co-IP, and the interaction domains of ACLY were detected via Myc antibody.

To identify proteins that might be regulated by USP13, we conducted immunoprecipitation-based mass spectrometry (IP-MS) following USP13 overexpression and subsequent immunoprecipitation. This analysis revealed that ACLY was among the proteins immunoprecipitated by USP13 (Fig. 4A). The ACLY protein is involved in amino acid metabolism and participates in methylation reactions, potentially contributing to the regulation of cellular metabolic equilibrium [33, 34]. Subsequent co-immunoprecipitation analysis demonstrated that endogenous USP13 was capable of co-precipitating with endogenous ACLY (Fig. 4B). To examine their cellular distribution, we performed immunofluorescence, which revealed that USP13 and ACLY were colocalized in the cytoplasm of HCC cells (Fig. 4C). Furthermore, to explore the precise regions responsible for the interaction between USP13 and ACLY, we conducted deletion analyses. As demonstrated in Fig. 4D–F, the USP and UBA domains of USP13 physically associate with ACLY. Likewise, we discovered that ACLY’s CoA ligase domain binds to USP13 (Fig. 4E–G).

### USP13 stabilizes ACLY through the deubiquitylation activity

**Fig. 5:**
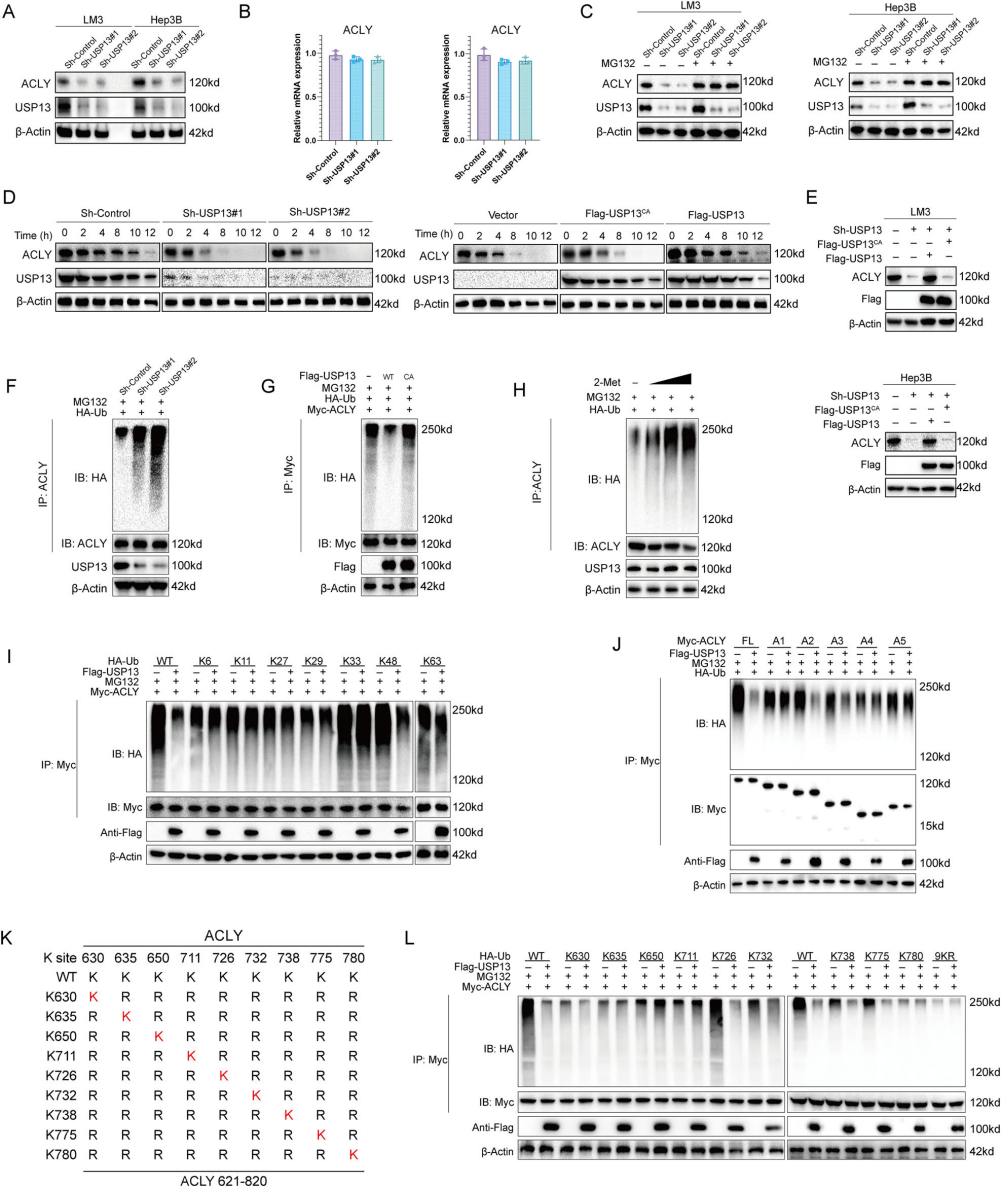
USP13 stabilizes ACLY through the deubiquitylation activity. **A**, **B** Knockdown of USP13 resulted in a reduction of ACLY protein levels, while the mRNA expression of ACLY remained unchanged. **C** ACLY protein levels were analyzed in USP13-depleted LM3 and Hep3B cells treated with or without the proteasome inhibitor MG132. **D** The half-life of ACLY was analyzed in cells transfected with the corresponding siRNA or plasmids. **E** The C345A mutant or USP13 WT was transfected into USP13-depleted LM3 and Hep3B cells, after which ACLY levels were quantified. **F** Ubiquitination of ACLY was analyzed in USP13-depleted LM3 cells. **G** Ubiquitination of ACLY was analyzed by immunoblotting in HEK293T cells co-transfected with HA-tagged ubiquitin, along with Flag-USP8 WT or the C345A mutant. **H** Ubiquitination of ACLY was analyzed in LM3 cells treated with or without 2-Met. **I** ACLY ubiquitination was analyzed in HEK293T cells transfected with HA-tagged wild-type or mutant ubiquitins. **J** The ubiquitination of ACLY mutants was analyzed by immunoblotting. **K** A schematic overview depicting ACLY and its corresponding mutants. **L** The ubiquitination of ACLY mutants in HEK293T cells was analyzed using immunoblotting.

Given that USP13 belongs to the ubiquitin-specific protease family, we speculated that USP13 might control ACLY degradation via the ubiquitin-proteasome pathway. Our experiments revealed that USP13 depletion significantly reduced ACLY protein levels, while its mRNA levels remained unchanged (Fig. 5A, B), and this reduction was reversed by treatment with MG132, a proteasome inhibitor (Fig. 5C). To confirm the impact of USP13 on ACLY stability, we treated cells with cycloheximide (CHX), a protein synthesis inhibitor. Depletion of USP13 in LM3 cells resulted in a shorter half-life for ACLY, while overexpression of USP13-WT in 293T cells enhanced its stability, and this effect was not observed in cells expressing USP13-C345A (Fig. 5D). Consistently, USP13 led to a marked reduction in ACLY levels, which could be restored by overexpression of wild-type USP13 (WT), but not its catalytically inactive mutant (USP13-C345A) (Fig. 5E). These findings suggest that USP13 maintains ACLY stability through the ubiquitin-proteasome pathway.

To determine if ACLY is a direct substrate of USP13, we examined its ubiquitination status. We found that USP13 depletion significantly elevated the ubiquitinated form of ACLY (Fig. 5F). In contrast, overexpression of USP13-WT, but not its inactive mutant USP13-C345A, led to a substantial decrease in ACLY ubiquitination (Fig. 5G). Consistently, inhibition of USP13 with 2-Met resulted in a dose-dependent increase in ACLY ubiquitination (Fig. 5H). Furthermore, in order to identify which type of ubiquitin chain is deubiquitylated by USP13 on ACLY, we performed a ubiquitination assay with several ubiquitin mutants (K6, K11, K27, K29, K33, K48, and K63). The findings showed that USP13 efficiently cleaved K48-linked ubiquitin chains from ACLY (Fig. 5I).

To further explore which ubiquitination site of ACLY is regulated by USP13, a ubiquitination assay was performed using different ACLY constructs (A1, A2, A3, A4, A5, and full-length) and USP13. The findings revealed that USP13 specifically targeted the CoA ligase domain of ACLY for polyubiquitin chain cleavage, without affecting the other domains (Fig. 5J). Using three bioinformatics tools (UbiSite, BDM-PUB, and UbPred), we predicted nine lysine residues in the CoA ligase domain of ACLY as potential ubiquitination sites. To pinpoint the specific sites targeted by USP13 for deubiquitination, we generated mutations in these lysines (Fig. 5K). Ubiquitination assays revealed that K726 is the key residue deubiquitinated by USP13 on ACLY (Fig. 5L).

### Hypoxia-induced USP13 expression promotes HCC progression through ACLY

**Fig. 6:**
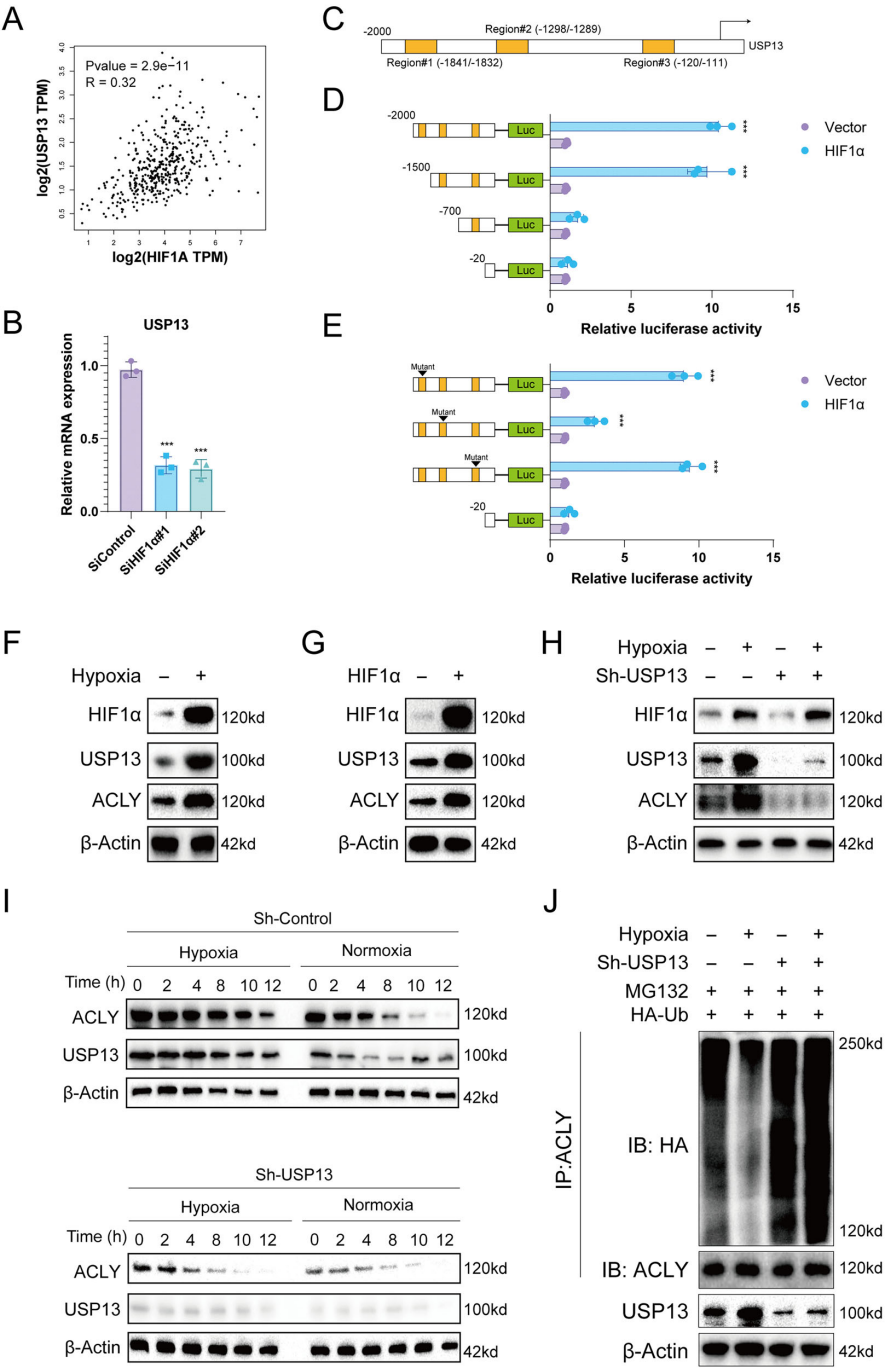
Hypoxia-induced expression of ACLY is dependent on USP13. **A** TCGA data analysis shows a strong positive correlation between HIF-1α and USP13. **B** Knockdown of HIF-1α in LM3 cells reduced USP13 levels. **C** Three HIF-1α binding sites were identified in the USP13 promoter via bioinformatics analysis. **D** Luciferase assays with truncated USP13 promoter plasmids confirmed HIF-1α binding to Region #2 of the USP13 promoter. **E** Mutating the USP13 promoter regions confirmed that HIF-1αcontrols its activity through Region #2 of the USP13 promoter. **F**, **G** Hypoxia or HIF-1α overexpression induces upregulation of USP13 and ACLY. **H** Hypoxia-induced ACLY upregulation is lost with USP13 knockdown. **I** ACLY stability is enhanced by hypoxia, but USP13 knockdown negates this effect. **J** Hypoxia reduces ACLY ubiquitination, but this is reversed by USP13 silencing, indicating USP13’s role in hypoxic regulation of ACLY. Data are expressed as mean ± SD based on triplicate biological samples. Statistical significance is defined as *p < 0.05, **p < 0.01, and ***p < 0.001.

Our research demonstrates that USP13 is highly expressed in liver cancer; however, the underlying cause of this elevated expression remains unclear. Numerous studies have reported that hypoxia plays a critical role in the onset and progression of liver cancer. Consistent with these findings, our analysis of TCGA data reveals a strong positive correlation between the hypoxia-inducible factor HIF-1α and USP13 (Fig. 6A). Furthermore, silencing HIF-1α expression in HCC cells resulted in a corresponding reduction in USP13 levels (Fig. 6B). Through bioinformatics analysis, we identified 3 binding sites for HIF-1α within the promoter region of USP13 (Fig. 6C). To further investigate this, we constructed luciferase reporter plasmids containing various truncated regions of the USP13 promoter. Luciferase assays revealed that HIF-1α specifically binds to Region #2 of the USP13 promoter (Fig. 6D). Additionally, we introduced mutations into different regions of the USP13 promoter and generated corresponding luciferase reporter plasmids. The results confirmed that HIF-1α regulates USP13 promoter activity by binding to the Region #2 site (Fig. 6E). Moreover, Hypoxic conditions or overexpression of HIF-1α can induce the upregulation of both USP13 and ACLY (Fig. 6F, G). Notably, the hypoxia-induced upregulation of ACLY is abolished when USP13 expression is knocked down (Fig. 6H). Furthermore, we observed that hypoxia increases ACLY stability, an effect that is lost upon USP13 knockdown (Fig. 6I). Under hypoxic conditions, the ubiquitination level of ACLY decreases, but this reduction is reversed when USP13 is silenced (Fig. 6J). We further performed rescue experiments by overexpression of exogenous ACLY in USP13-deficient cells. We observed that exogenously expressed ACLY reversed the anti-tumor effects induced by USP13 depletion (Fig. S6). These findings suggest that hypoxia-induced USP13 expression promotes HCC progression through ACLY.

## Discussion

HCC is a highly aggressive malignancy with a poor prognosis[1, 35]. Recent advances in understanding the genetic and molecular mechanisms underlying HCC have opened new avenues for targeted therapies. Among these, DUBs have garnered increasing attention for their role in cancer initiation and progression [8, 36]. DUBs regulate various cellular processes, including protein degradation, DNA repair, and signal transduction, by modulating ubiquitination status [9, 37, 38]. Small molecule DUB inhibitors are emerging as promising candidates for novel anticancer therapies [39–41], but their potential role in HCC treatment remains unclear, warranting further investigation into their therapeutic mechanisms and efficacy.

USP13 has been found to be highly expressed in various tumors, including lung, kidney, and cervical cancers [42–44]. Its high expression is often associated with increased tumor cell proliferation and enhanced cancer stemness [45, 46]. Recent studies have explored the role of USP13 in modulating the tumor immune microenvironment, highlighting its influence on immune evasion mechanisms, such as regulating immune checkpoint pathways and immune cell activity [47, 48]. USP13 has been implicated in the stabilization of PD-L1, a critical immune checkpoint molecule that helps tumors escape immune detection. Inhibiting USP13 has been shown to decrease PD-L1 levels, potentially enhancing the effectiveness of immune checkpoint inhibitors like anti-PD-1 therapy [49]. In addition, USP13 was reported to be involved in protein neddylation and is associated with immune cell infiltration and immunomodulator expression. Targeting USP13 could potentially inhibit tumor growth and enhance the effectiveness of immunotherapy [50]. Combining USP13 inhibitors with existing immunotherapies, such as PD-1/PD-L1 inhibitors, represents a promising strategy to improve anti-tumor efficacy. This combination approach may enhance immune responses by simultaneously reducing tumor immune evasion and promoting immune cell activity within the tumor microenvironment.

In this study, we observed that pharmacological inhibition or knockdown of USP13 significantly suppressed the progression of HCC. We first assessed the anti-cancer effect of the USP13 inhibitor 2-Met in HCC cells. The results demonstrated that inhibition of USP13 reduced cell proliferation, colony formation, and stemness in HCC cells, while also impairing cholesterol metabolism and inducing ferroptosis. To explore the underlying mechanisms, we performed LC-MS-based metabolomics analysis. Interestingly, treatment with 2-Met led to substantial alterations in the cholesterol metabolism pathway. Cholesterol metabolism produces essential membrane components as well as metabolites with a variety of biological functions. Cholesterol biosynthesis starts with acetyl-CoA in the mevalonate pathway. In this process, the key regulatory rate-limiting glycoprotein enzyme, 3-hydroxy-methylglutaryl-CoA reductase, located in the endoplasmic reticulum, converts HMG-CoA to mevalonate using NADPH [51]. Dysregulation of cholesterol homeostasis is associated with various diseases in humans, including inflammatory diseases, neurodegenerative disorders, cardiovascular diseases, and cancers. In the tumor microenvironment, intrinsic and extrinsic cellular factors reprogram cholesterol metabolism and consequently promote tumorigenesis. Ferroptosis is a non-apoptotic form of regulated cell death characterized by the lethal accumulation of iron-dependent membrane-localized lipid peroxides [52, 53]. Extensive studies suggest that ferroptosis plays a pivotal role in tumor suppression, thus providing new opportunities for cancer therapy [54]. Previous studies have shown a close relationship between cholesterol metabolism and ferroptosis. The inhibition of cholesterol uptake sensitizes certain cancer cell lines to ferroptosis [55–59]. For instance, Liu W. et al. demonstrated the impact of cholesterol homeostasis on cancer cells’ resistance to ferroptosis and its correlation with increased tumor growth and metastasis [55]. Mechanistically, Cholesterol alters metabolic flux of the mevalonate pathway by promoting Squalene Epoxidase degradation, a rate limiting step in cholesterol biosynthesis, thereby increasing both CoQ and squalene levels [60]. As a consequence, the elevated levels of squalene and CoQ protect cells from ferroptosis [61]. 7-dehydrocholesterol dictates ferroptosis surveillance by using the conjugated diene to exert its anti-phospholipid autoxidation function and shields plasma and mitochondria membranes from phospholipid autoxidation [62]. Cholesterol can also increase the stiffness of the plasma membrane and thereby alleviate the propagation of lipid peroxidation. In addition, cholesterol has been found to be correlated with GPX4 expression in cholesterol auxotrophic cells [63, 64]. The role of cholesterol metabolism in regulating ferroptosis has been extensively studied, suggesting that pharmacological manipulation of cholesterol levels is a promising therapeutic strategy for cancer treatment. A recent study revealed that USP13 interacts with and catalyzes the deubiquitination of the transcription factor NFE2L2 and promotes an autophagy-to-ferroptosis switch in KRAS mutant lung adenocarcinoma [65]. Our study demonstrated that targeting USP13 promoted ferroptotic cell death triggered by the ferroptosis inducers. And we observed that GSH levels were significantly decreased in 2-Met-treated or USP13-deficient cells, while ROS accumulated in these cells. This highlights the potential of targeting USP13 in HCC to modulate redox homeostasis and induce ferroptosis, presenting a novel therapeutic approach.

Several studies have reported that cholesterol metabolism in tumors can affect the function of T cells within the tumor microenvironment, impacting their ability to mount an effective antitumor response [32, 66, 67]. Cholesterol drives CD8 + T cell exhaustion by inducing the expression of immune checkpoints through the activation of XBP1. Reducing cholesterol restores antitumor T cell function, offering a potential strategy to improve T cell-based immunotherapy [32]. Increased cholesterol levels were negatively associated with CD8 + T cell infiltration and function and predicted poorer outcomes in patients with CCA undergoing neoadjuvant chemoimmunotherapy [68]. Yang W et al. reported that enhancing the antitumor response of CD8(+)T cells can be achieved by modulating cholesterol metabolism [69]. GZMB (Granzyme B) is a serine protease released by cytotoxic T cells and natural killer (NK) cells. It plays a key role in inducing apoptosis in target cells by cleaving substrates within the cytoplasm, leading to cell death [70, 71]. IFN-γ (Interferon Gamma) is a cytokine produced by activated T cells and NK cells. It is crucial for immune surveillance and defense against infections and tumors. IFN-γ promotes antigen presentation and activates macrophages, making it an essential indicator of T cell activity in immune responses [72, 73]. Both GZMB and IFN-γ serve as important indicators of T cell activity in immunological contexts, especially in antitumor responses [74, 75]. Our research found that treatment with 2-Met or deletion of USP13 in tumor cells led to significantly increased CD8 + T cell infiltration, along with elevated GZMB and IFN-γ secretion, indicating enhanced T cell activity. Additionally, in vivo experiments using a xenograft mouse model revealed that inhibition of USP13 notably reduced tumor growth, while increasing CD8 + T cell infiltration and boosting GZMB and IFN-γ secretion in tumor tissues, thus enhancing T cell activity. We further found that cholesterol supplementation reversed USP13 depletion-induced T cell function enhancement, while M-β-CD, a cholesterol-depleting agent, nearly abolished USP13’s regulatory effect on CD8 + T cells. These findings suggest that USP13 plays a key role in immune responses of tumors through modulating cholesterol metabolism.

ATP citrate lyase (ACLY) is a key enzyme involved in the metabolic reprogramming of cancer cells, as it catalyzes the conversion of citrate to acetyl-CoA, a precursor for lipid synthesis. This process is critical for producing membrane lipids and acetylation reactions that support rapid tumor growth [33, 34, 76]. ACLY is often upregulated in various cancers, and its inhibition has emerged as a potential target for cancer therapy [77, 78]. ACLY has been shown to be relevant in modulating immune cell function [79, 80]. For example, Vaughn N et al. demonstrated the critical role of ACLY in early CD8 + T cell activation, impacting both metabolic processes and histone acetylation, particularly through regulating the transcription factor IRF4 [81]. Another study highlights the role of ACLY in regulating immune responses, both innate and adaptive, by modulating lipid metabolism and epigenetic changes in immune cells [79]. These findings suggest that targeting ACLY could improve the effectiveness of immunotherapy by altering the tumor microenvironment and enhancing immune cell activity. In our study, we found that USP13 functions as an effective deubiquitinating enzyme responsible for the deubiquitination and stabilization of ACLY. First, the interaction between USP13 and ACLY was confirmed through co-immunoprecipitation analysis. Second, USP13 promotes ACLY protein stability by reducing its polyubiquitination, a process dependent on the deubiquitination activity of USP13. USP13 significantly decreased K48-linked polyubiquitination of ACLY, which typically leads to proteasomal degradation of proteins. The loss of USP13 markedly increased ACLY ubiquitination levels and reduced ACLY protein levels, an effect that could be reversed by overexpressing (WT), but not by the catalytically inactive mutant USP13 C345A, further revealed that K726 is the key site on ACLY targeted by USP13 for deubiquitination. Additionally, we discovered that the high expression of USP13 in HCC is closely related to hypoxic conditions. Hypoxia-induced upregulation and stabilization of ACLY were dependent on USP13, and the knockdown of USP13 reversed these effects. Under hypoxic conditions, HIF-α promotes USP13 transcription by binding to its promoter, thereby increasing ACLY expression and stability. This highlights the potential therapeutic value of targeting USP13 and ACLY in cancer treatment.

**Fig. 7:**
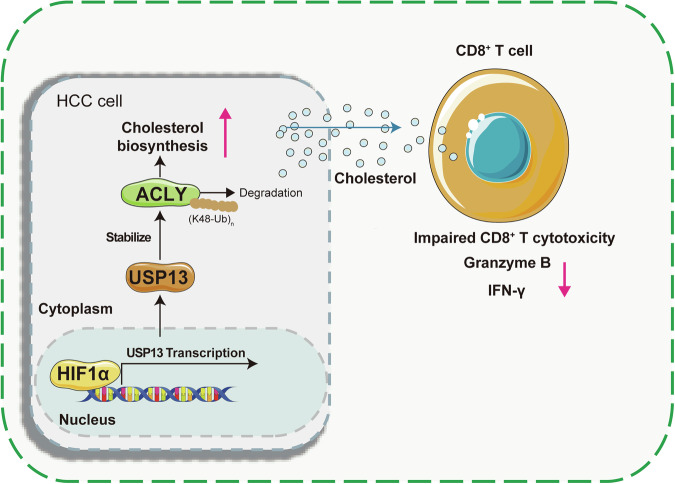
Graphic summary of HIF-1α-USP13-ACLY pathway. HIF-1α activates USP13 transcription by binding to its promoter. The elevated USP13 deubiquitinates and stabilizes ACLY by removing K48-linked ubiquitin chains. The activation of HIF-1α-USP13-ACLY pathway drives ferroptosis resistance and tumor immune evasion in hepatocellular carcinoma.

In summary, we demonstrated that targeting USP13 inhibits cholesterol metabolism of HCC, thus inducing ferroptosis and promoting CD8 + T cell cytotoxicity. Mechanistically, the expression of USP13 is upregulated by HIF-1α. The elevated USP13 deubiquitinates and stabilizes ACLY by removing K48-linked ubiquitin chains (Fig. 7). Our findings provide new insights into the role of USP13 in regulating ferroptosis and T cell function, and suggest that targeting USP13 is a promising therapeutic strategy for HCC.

## Materials and methods

We obtained scRNA-seq data of liver cancer samples (GSE125449, GSE149614, GSE151530, GSE156625, GSE162616, GSE166635, GSE189903, GSE202642, GSE210679, GSE242889, GSE282701) from Gene Expression Omnibus (GEO). ScRNA-seq data were initially filtered by the R package Seurat, and standard Seurat settings were employed for normalization, principal component analysis. Furthermore, the R package Harmony was utilized to remove batch effects between cohorts. Cell visualization was performed using t-distributed stochastic neighbor embedding analysis. The cell annotations were primarily based on previous studies.

### Cell culture

LM3 and Hep3B human liver cancer cells, along with HEK293T human embryonic kidney cells, were purchased from Procell Life Science & Technology Co., Ltd. (Wuhan, China). Cell authentication was performed via short tandem repeat analysis by the respective cell banks. All cells were cultured in Dulbecco’s modified Eagle’s medium (DMEM, 41965, Life Technologies) with 10% fetal bovine serum (FBS, Gibco, Life Technologies, 10270) and incubated at 37 °C in a 5% CO2 humidified environment. PDOs from fresh HCC samples using organoid culture kit according to the manufacturer’s protocols (Absin, China) as reported in our previous study [82]. HCC organoids were dissociated using Organoid Digestion Solution (abs9520, China) and plated at a density of 2000 cells in 50 μL matrigel droplets in order to form organoids and grown in Human Hepatocarcinoma Organoid Culture Medium (abs9527, China) in 96-well plates. After 3 days, 10 μM of each compound was added to the culture medium, and cell viability was assayed using CellTiter-Glo (Promega) after 48 h of drug incubation according to the manufacturer’s instructions.

### Plasmids and establishment of stable expression cell lines

Plasmids for ACLY, USP13, and their mutants were sourced from Hanbio Biotechnology Co., Ltd. (Shanghai, China), while the HA-tagged K6, K11, K27, K29, K33, K48, K63, and Ub plasmids were obtained from Addgene. To created stable USP13-knockdown cells, PLVX-shUSP13-Puro (sh-USP13#1: CGATTTAAATAGCGACGATTA and sh-USP13#2: GCCAGTATCTAAATATGCCAA), were synthesized by Genepharma (Shanghai, China). HEK293 cells were transfected with the PLVX-shUSP13-Puro plasmids, along with the packaging plasmids psPAX2 and pMD2, using Lipofectamine 2000. Viral supernatants were harvested 24 and 48 h post-transfection and filtered through a 0.45 μm membrane to remove cell debris. Following a 24-h infection with the lentivirus, HCC cells were selected and maintained with 1 μg/mL puromycin.

### Co-immunoprecipitation analysis

Following a pre-chilled PBS wash, cells were lysed with RIPA buffer (Meilun, China) containing protease inhibitors (Meilun, China). After pre-clearing, cell lysates were incubated overnight at 4 °C with the specified antibody. The antibody-bound protein complexes were captured using protein A/G PLUS-Agarose beads for a further 2 h. Afterward, the beads were rinsed 3 times with PBS and boiled at 100 °C for 10 min to break the crosslinking, and subsequently subjected to SDS-PAGE and immunoblotting.

### Protein stability evaluation

For measuring ACLY half-life, cells were incubated with 100 μM CHX (Sigma Aldrich), a protein synthesis inhibitor, for the specified time points. ACLY protein levels were evaluated through Western blotting.

### Western blotting assay

Proteins were extracted with NP-40 lysis buffer containing protease inhibitors (Meilun, China), and protein concentration was determined using the BCA assay (Thermo Scientific, Rockford, IL, USA). The extracted proteins were separated on an SDS-polyacrylamide gel and transferred onto a 0.45 μm PVDF membrane (Millipore, USA). The following primary antibodies were employed: ACLY (Proteintech, 15421-1-AP), USP13 (Proteintech, 16840-1-AP and 66176-1-Ig), β-Actin (Proteintech, 66009-1-Ig), Flag (Proteintech, 66008-4-Ig), Myc (Proteintech, 60003-2-Ig), and HA (Proteintech, 510642-AP). ECL detection (Meilun, China) was used to visualize the signals, followed by imaging on a ChemiDoc MP system (Bio-Rad).

### Immunohistochemistry (IHC) analysis

The liver cancer tissues and corresponding adjacent tissues were acquired from the Department of Liver Surgery at Xiangya Hospital and were validated by two pathologists independently. The tissues were embedded in paraffin for sectioning (4 μm continuous sections), fully baked, and placed in citrate buffer for 10 min. The sections were incubated with monoclonal antibodies against USP13 overnight at 4 °C and then treated with the secondary antibody at room temperature (RT) for 30 min. USP13 staining was evaluated under a microscope. Staining result and evaluation: We randomly selected 5 fields of view and scored the colored parts of the cells: unstained cells were scored 0; light yellow cells were scored 1, dark yellow cells were scored as 2, and brown cells were scored as 3. According to the score results, the samples were divided into 2 groups: a score of <2 was evaluated as being negative and was set as a low-expression level sample; a score of 2 was evaluated as positive, with a high expression level. The research was approved by the Ethics Committee at Xiangya Hospital of Central South University (Approval number: 2025040650).

### Analysis of deubiquitination in vivo

HEK293T cells were transfected with HA-Ub, Myc-ACLY, Flag-USP13, or Flag-USP13 C345A plasmids for 48 h. Following transfection, cells were treated with 10 μM MG132 (MCE) for 6 h. The cells were then washed with ice-cold PBS and lysed using RIPA buffer. Immunoprecipitation of HA-ubiquitinated ACLY was carried out using an anti-Myc antibody, followed by detection of ACLY ubiquitination through Western blotting with an anti-HA antibody. Moreover, USP13-knockdown LM3 HCC cells were transfected with the HA-Ub plasmid. HA-ubiquitinated ACLY was pulled down using an anti-ACLY antibody, and the ubiquitination levels of ACLY were subsequently detected by Western blotting with an anti-HA antibody.

### Lipid ROS detection

Cells were first washed with PBS, followed by incubation with 10 μM C11-BODIPY (581/591) (Thermo Fisher Scientific, #D3861) in PBS at 37 °C for 30 min. The excess dye was removed by washing the cells again with PBS, and the cells were harvested for flow cytometry analysis (Fortessa, BD Biosciences) using the fluorescein isothiocyanate (FITC) green and Texas red fluorescence channels.

### GSH level analysis

The detection of total GSH was performed using the Glutathione Assay Kit (Beyotime Biotechnology) following the manufacturer’s protocol. In short, HCC cells were washed with PBS, collected into centrifuge tubes, and immediately treated with three volumes of protein removal reagent S. After vigorous vortexing, the samples were subjected to two freeze-thaw cycles between liquid nitrogen and 37 °C water. After standing at 4 °C for 5 min, the samples were centrifuged at 10,000 × *g* for 10 min at 4 °C, and the resulting supernatant was used for GSH quantification.

### Animal experiments

The animal experiments followed the ethical guidelines approved by the Xiangya Hospital Ethics Committee. 4-week-old C57BL/6 mice were obtained from Beijing HFK Bioscience Co., Ltd. (Beijing, China). When the mice reached 6 weeks of age, Hepa1-6 cells (1 × 10^6) suspended in 100 μL of DMEM were injected subcutaneously into the flanks. Tumor size was measured using a vernier caliper every other day throughout the experiment. For DEN-induced hepatocarcinoma model, 14-day-old C57BL/6 male mice were intraperitoneally injected with diethylnitrosamine (DEN, dissolved in PBS, 25 mg/kg body weight). Mice were sacrificed for analysis at 28 weeks. Mice were intraperitoneal injected with 2-Met (50 mg/kg per 2 days) for 12 weeks.

### Cell viability/death staining

Cells were stained to distinguish between viable and non-viable populations using Calcein-AM for live cells and Propidium Iodide (PI) for dead cells. Following a PBS wash, treated cells were incubated at 37 °C for 15 min with 2 μM Calcein-AM and 2 μM PI (Meiun, China). After incubation, a fluorescence microscope (N2-DMi8, Leica) was used to obtain images of the stained cells.

### Flow cytometry analysis

Tissue samples were dissociated into single cells with RPMI 1640, and red blood cells (RBCs) were removed by RBC lysis buffer. After filtering through a 70 μm cell strainer, cells were resuspended in staining buffer. Then, cells were incubated with Fixable Viability Stain 510 (BD Biosciences, # 564406) for 15 min at RT, followed by incubated with Fc Receptor Blocking Solution (BD Biosciences, #553142) for 15 min at 4 °C and stained with anti-CD45-APC-Cy7(BD Biosciences, #557659, 1:200), anti-CD8-PerCP-Cy5.5 (eBiosciences, 45-0081-82, 1:200), anti-CD4-FITC (BD Biosciences, #553729, 1:200), anti-CD3e-APC (BD Biosciences, #553066, 1:200), anti-Granzyme B-PE (Biolegend, #372208, 1:200), anti-IFN-γ-PE-Cy7 (eBiosciences, 25-7311-82, 1:200) for 30 min at 4 °C in dark. After washing, samples were detected with a Fortessa flow cytometer (BD Biosciences, NJ) and data were analyzed with FlowJo V10.

### T cell co-culture assay

For T cell coculture assay, human peripheral blood T cells were isolated by the human CD3 T cell Isolation Kit (Biolegend, 480131). Isolated T cells were activated with human T-activation CD3/CD28 Dynabeads (Invitrogen) and human IL-2 (R&D Systems, 10 ng/ml) for 72 h. Then, activated T cells were added to the culture medium of HCC cells at a ratio of 5:1. At 72 h after co-incubation, tumor cells were collected and stained with Calcein-AM/PI or crystal violet. Quantification of IFN-γ or GZMB in supernatant was determined by ELISA.

### Cell proliferation assay

To assess cell proliferation, LM3 and Hep3B cells (5 × 10³) were plated in 96-well plates. After the cells adhered, the drugs were introduced, and cell proliferation was evaluated at designated time points using the CCK8 assay, with absorbance at 450 nm normalized to control wells to determine proliferation.

### Tumorsphere formation assay

Cells are cultured in a serum-free DMEM/F12 medium in ultra-low attachment plates. A combination of B27 (1:50), 20 ng mL−1 EGF, and 20 ng mL−1 bFGF is typically added to the medium. Cells are plated at a density of 2 × 10^3^ cells per well in ultra-low attachment 6-well plates, and after two weeks, spheres are counted using a microscope.

### Mass spectrometry analysis

The IP-MS technique was performed by OE Biotech Co., Ltd., and the detection procedure has been detailed in earlier studies [83]. For the metabolomics experiments, HCC cells were plated and treated with 2-Met (5 μM) or DMSO for a duration of 24 h. After scraping, cells were flash-frozen in liquid nitrogen and stored at −80 °C for extraction. Changes in metabolite levels were analyzed using a GC/TOF-MS-based metabolomics approach, as documented previously [84]. OE Biotech Co., Ltd. (Shanghai, China) carried out all data analysis.

### Statistical analysis

Prism 9.5 software (GraphPad, USA) was used to carry out statistical analysis, including two-tailed Student’s *t* tests or ANOVA where applicable. Bonferroni’s correction was employed for multiple comparisons. Results from at least three independent experiments are expressed as mean ± standard deviation. Significance levels are denoted as follows: *p < 0.05, **p < 0.01 and ***p < 0.001.

## Supplementary information

Supplementary Figures

Western blot

## Data Availability

The data that support the findings of this study are available from the corresponding author upon reasonable request.
