# Supplementary material for: Hypoxia-induced USP13 expression drives ferroptosis resistance and tumor immune evasion in hepatocellular carcinoma through the stabilization of ACLY
Source: Cell Death Discov. 2025 Dec 2;12:28. doi: 10.1038/s41420-025-02869-z (PMC12811253; doi:10.1038/s41420-025-02869-z)
Supplement: Supplementary file 1 — Supplementary Figures [file 41420_2025_2869_MOESM1_ESM.pdf]

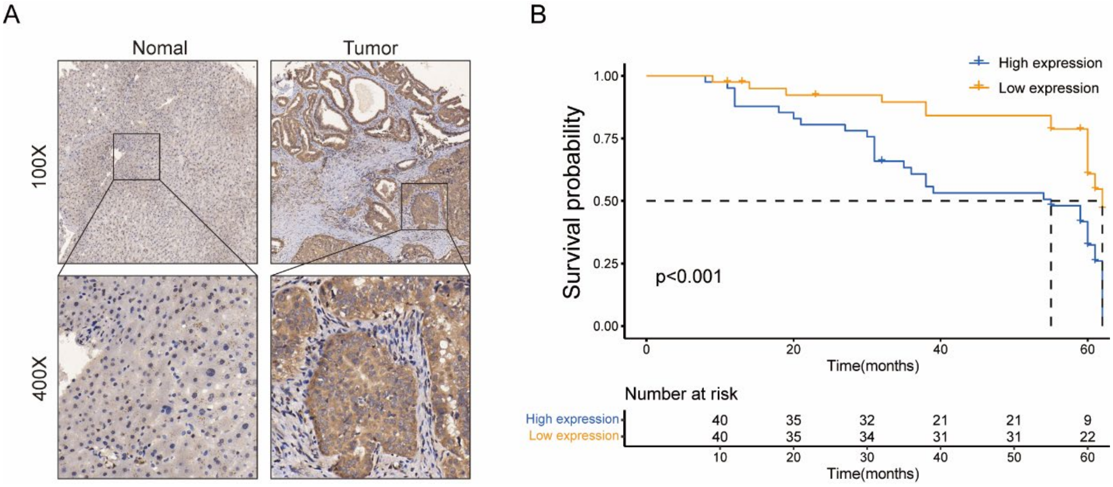

Supplementary Figure 1. Increased USP13 expression in HCC predicts poor prognosis. (A) Representative immunohistochemical staining images of USP13 show its significant overexpression in HCC tissues. (B) Survival analysis indicates that USP13 is associated with poorer prognosis, based on data from 80 HCC patients.

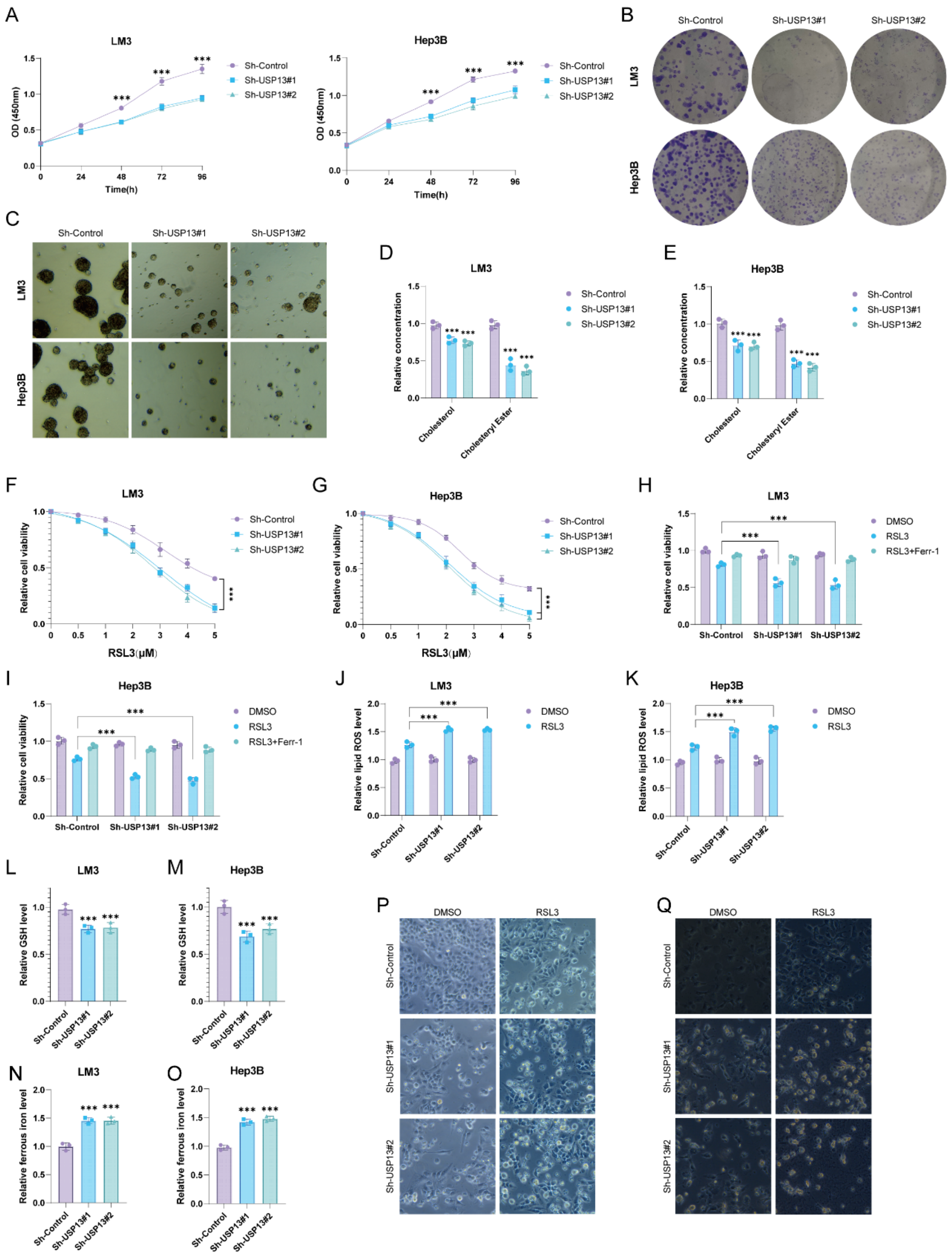

Supplementary Figure 2. USP13 depletion inhibits cell proliferation, stemness, and cholesterol metabolism while promoting ferroptosis. (A) CCK8 assays were performed on USP13-depletion LM3 and Hep3B cells. (B) Colony formation assays were performed on USP13-depletion LM3 and Hep3B cells. (C) Tumorsphere formation assays were performed on USP13-depletion LM3 and Hep3B cells. (D, E) The concentration of cholesterol and cholesteryl ester in USP13-depletion LM3 and

Hep3B cells were evaluated. (F, G) The viability of USP13-depletion LM3 and Hep3B cells were assessed as the RSL3 concentration was progressively increased (48 hours). (H, I) CCK8 assay was used to assess the response of USP13-depletion LM3 and Hep3B cells to RSL3 (5μM) with or without ferrostatin (1 μM). (J, K) Lipid ROS levels were measured in USP13-depleted LM3 and Hep3B cells, both with and without RSL3. (L, M) GSH levels were measured in USP13-depletion LM3 and Hep3B cells. (N, O) Ferrous iron levels were measured in USP13-depletion LM3 and Hep3B cells. (P, Q) Representative images of cell morphology were photographed in USP13-depletion LM3 and Hep3B cells, with and without RSL3 treatment. These results presented are representative of three independent experiments. Data are expressed as mean ± SD based on triplicate biological samples. Statistical significance is defined as *p < 0.05, **p < 0.01, and ***p < 0.001.

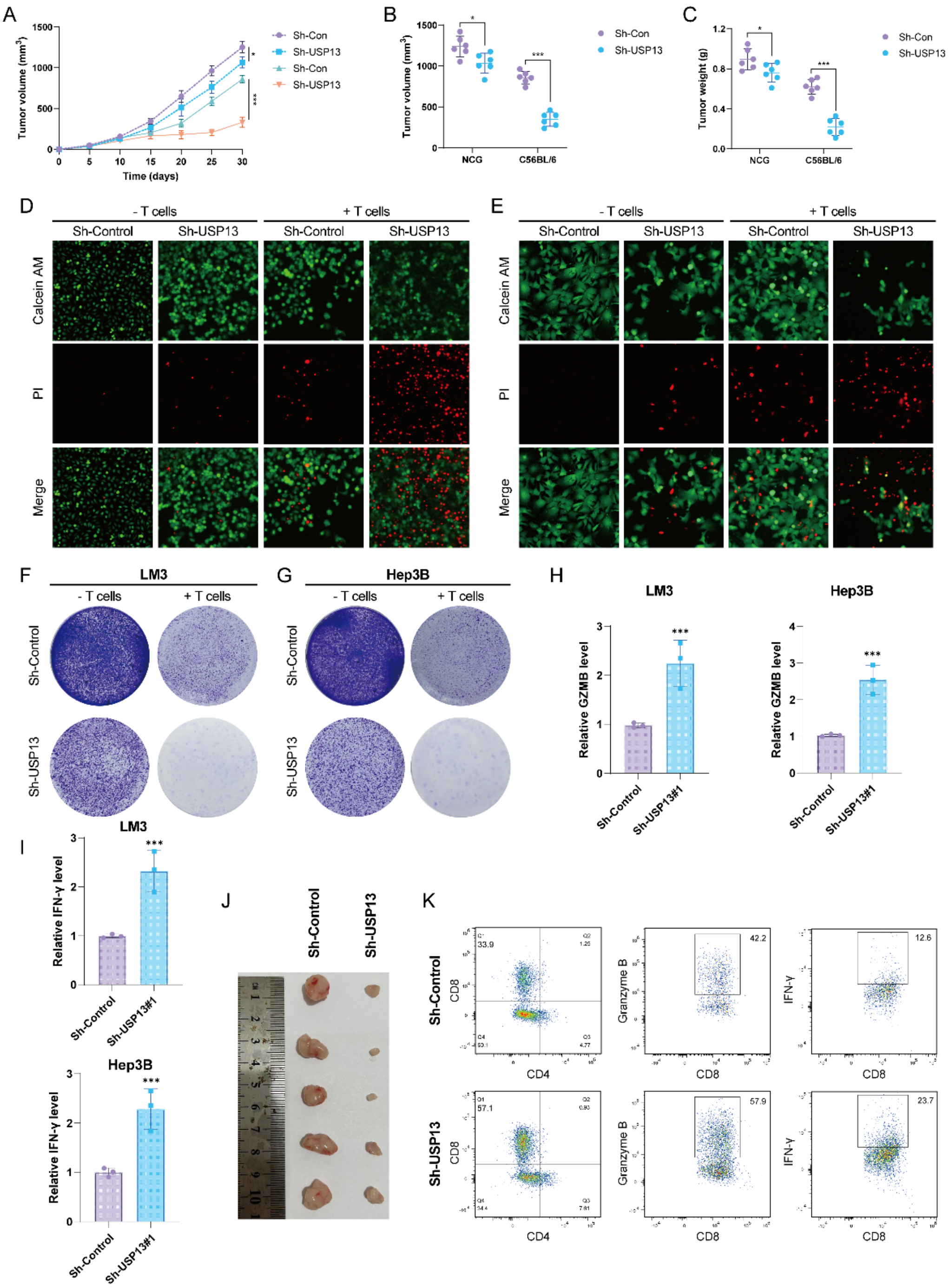

Supplementary Figure 3. Depletion of USP13 enhances T cell function in hepatocellular carcinoma. (A) Growth curve of C57BL/6 mice or NCG mice depleted with or without USP13. (B, C) Tumor volume and tumor weight at endpoint of C57BL/6 mice or NCG mice depleted with or without USP13. (D, E) T cells from human peripheral blood were co-cultured with HCC cells, with or without USP13 depletion. Calcein/PI staining indicated that more cell death in the T cells co-culture group, and USP13 depletion increased T cell-mediated tumor cell death. (F, G) T cells

from human peripheral blood were co-cultured with HCC cells, with or without USP13 depletion. Crystal violet staining assays indicated that 2-met treatment increased T cell-mediated tumor cell death. (H) GZMB levels were measured in supernatant determined by ELISA. (I) IFN-γ levels were measured in supernatant determined by ELISA. (J) C57BL/6 xenograft data show that USP13 depletion markedly inhibits HCC tumor growth in vivo. Mice harboring Hepa1-6 xenografts received control (n = 6) or USP13 depletion (n = 6). (K) Flow cytometric data indicated a marked increase in the population of infiltrating CD8+ T cells in tumors, coupled with upregulated GZMB+ and IFN-γ+ expression in these cells with USP13 depletion. These results presented are representative of three independent experiments. Data are expressed as mean ± SD based on triplicate biological samples. Statistical significance is defined as $^{*}p < 0.05$, $^{**}p < 0.01$, and $^{***}p < 0.001$.

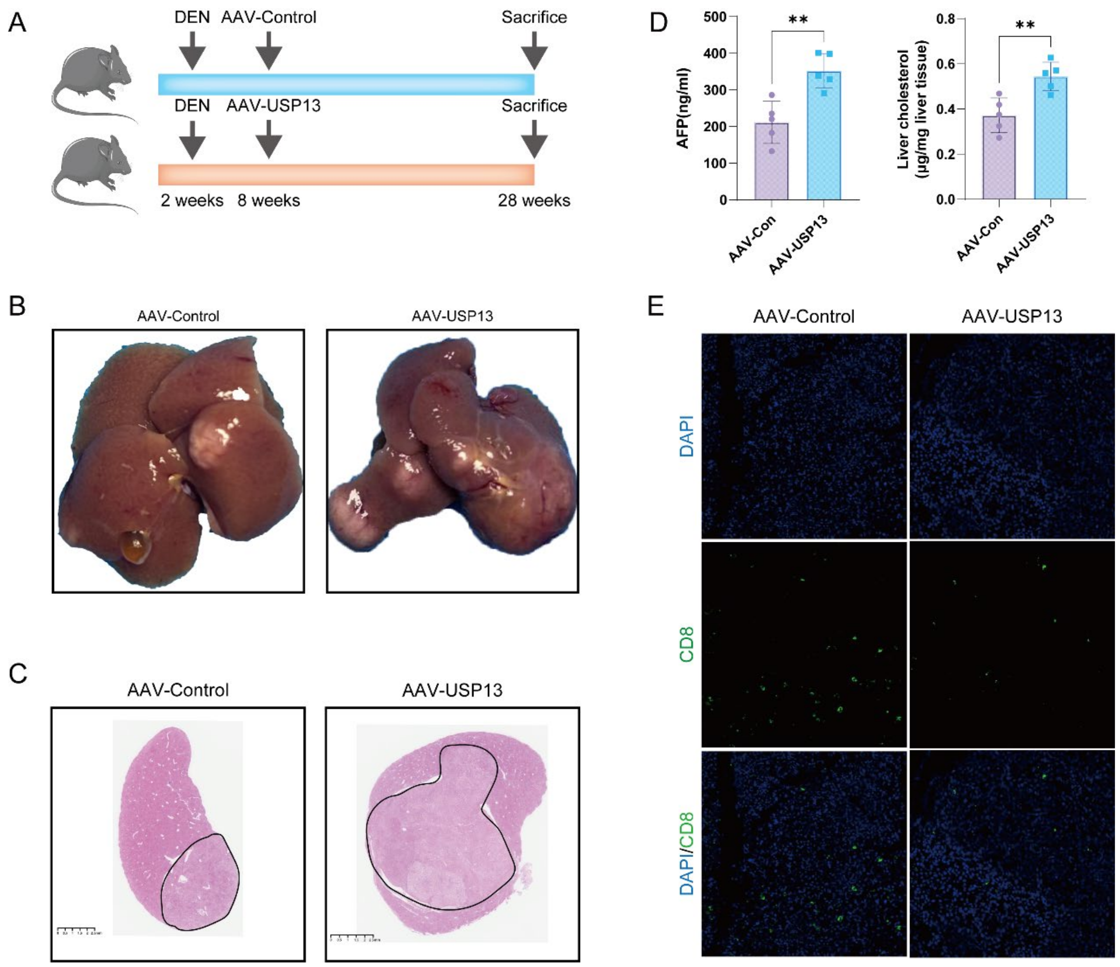

Supplementary Figure 4. The primary hepatocellular carcinoma model highlights the therapeutic efficacy of AAV-USP13. (A) The schematic diagram illustrating the administration of AAV-USP13 to nude mice. The mice were divided into two groups of six, concentrated AAV8-encoding USP13 or AAV8-control were delivered by portal vein injections. The mice were sacrificed at 28 weeks for liver collection. (B) Representative images of livers with tumors are presented, comparing the AAV-control group and the AAV-USP13 group. (C) Representative HE-stained microscopic images of liver tumors, comparing the AAV-control group and the AAV-USP13 group. (D) AFP levels were measured in nude mice from both the AAV-control group and the AAV-USP13 group. (E) Representative fluorescence microscopy images were used to assess the infiltration of CD8+ T cells in nude mice from the AAV-control group and the AAV-USP13 group.

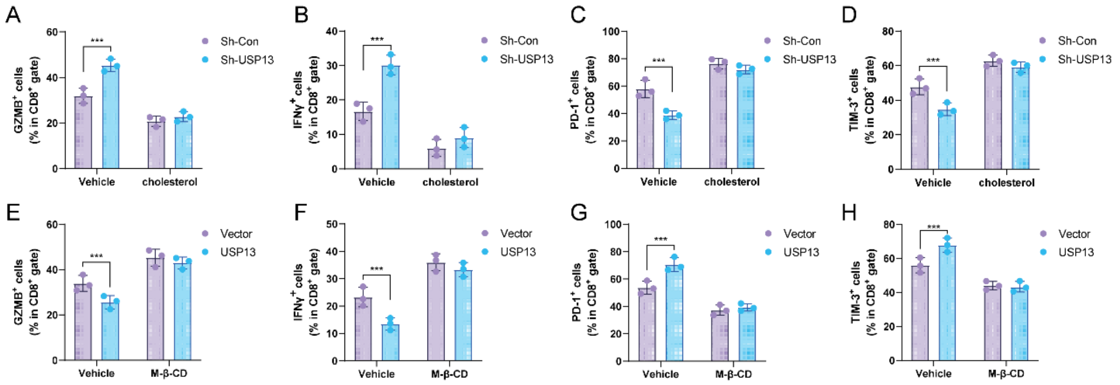

Supplementary Figure 5. USP13 promotes suppressed immune microenvironment of HCC by increasing free cholesterol. (A-D) Sh-Con and Sh-USP13 LM3 cells co-cultured with human CD8+ T cells, cholesterol (0.2 mg/mL) was added as indicated, followed by evaluation of T cell function and exhaustion levels. (E-F) Sh-Con and Sh-USP13 LM3 cells co-cultured with human CD8+ T cells, b-Cyclodextrin (b-CD, 0.5 mM) was added as indicated, followed by evaluation of T cell function and exhaustion levels. Data are expressed as mean ± SD based on triplicate biological samples. Statistical significance is defined as *$p < 0.05$, **$p < 0.01$, and ***$p < 0.001$.

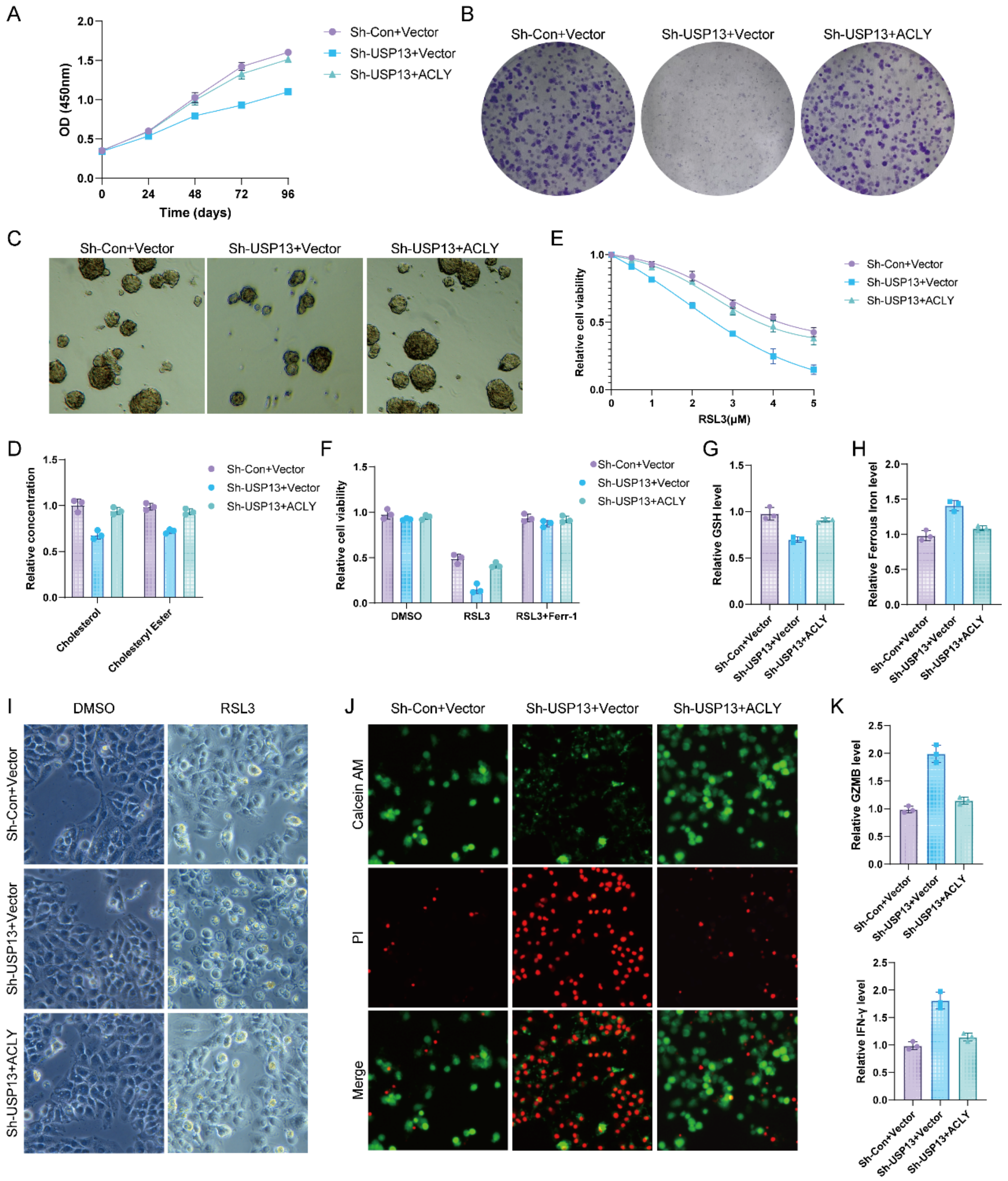

Supplementary Figure 6. USP13 promotes hepatocellular carcinoma progression through ACLY. (A, B) CCK-8 and colony formation assays were performed on LM3 cells following USP13 depletion and rescued with ACLY expression. (C) Tumorsphere formation assays were performed on LM3 cells following USP13 depletion and rescued with ACLY expression. (D) The concentration of cholesterol and cholesteryl ester in LM3 cells following USP13 depletion and rescued with ACLY expression. (E) The viability of LM3 cells following USP13 depletion and rescued with ACLY expression were assessed as the RSL3 concentration was progressively increased (48 hours). (F)

CCK8 assay was used to assess the response of LM3 cells to RSL3 (5μM) with or without ferrostatin (1 μM). (G, H) GSH and ferrous iron levels were measured in LM3 cells following USP13 depletion and rescued with ACLY expression. (I) Representative images of cell morphology were photographed in LM3 cells following USP13 depletion and rescued with ACLY expression, with and without RSL3 treatment. (J) T cells from human peripheral blood were co-cultured with LM3 cells following USP13 depletion and rescued with ACLY expression. Calcein/PI staining indicated that more cell death in the T cells co-culture group, and USP13 depletion increased T cell-mediated tumor cell death. (K) GZMB and IFN-γ levels were measured in supernatant determined by ELISA. Statistical significance is defined as $*p < 0.05$, $**p < 0.01$, and $***p < 0.001$.
