# Supplementary material for: Hypoxia-induced USP13 expression drives ferroptosis resistance and tumor immune evasion in hepatocellular carcinoma through the stabilization of ACLY
Source: Cell Death Discov. 2025 Dec 2;12:28. doi: 10.1038/s41420-025-02869-z (PMC12811253; doi:10.1038/s41420-025-02869-z)

Figure 4B

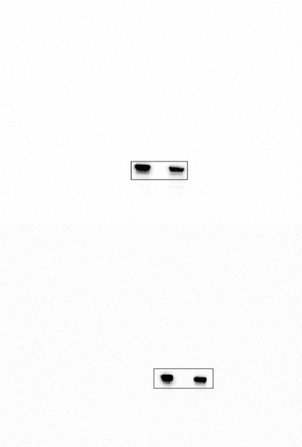

Figure 4F

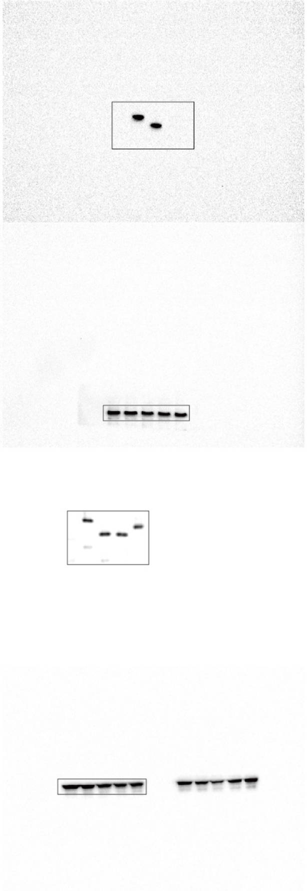

Figure 4G

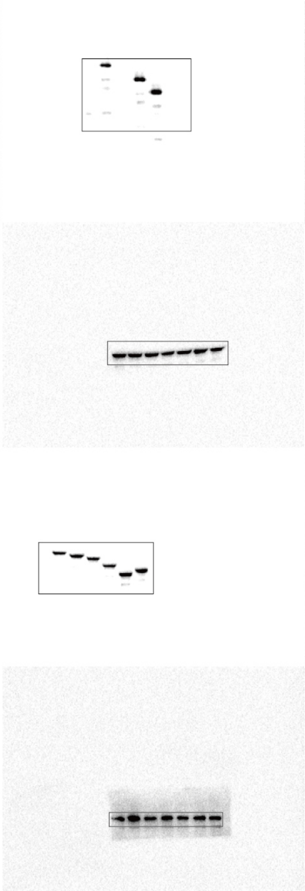

Figure 5A

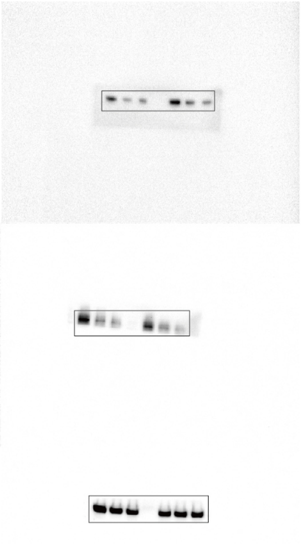

Figure 5C

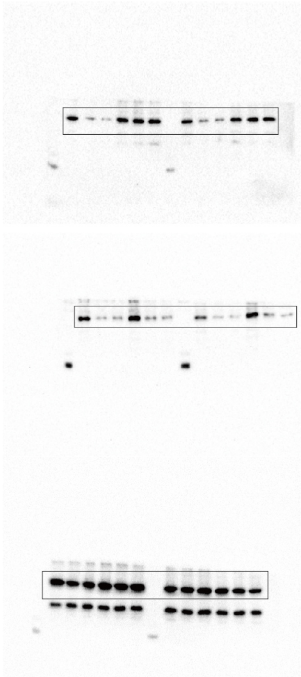

Figure 5D

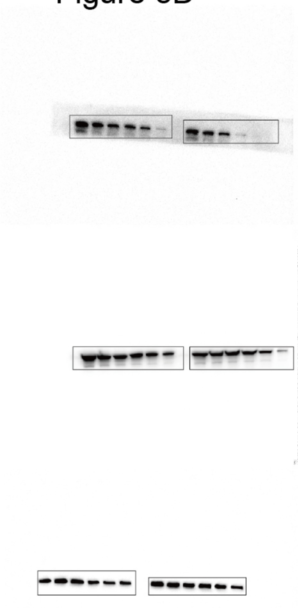

Figure 5D

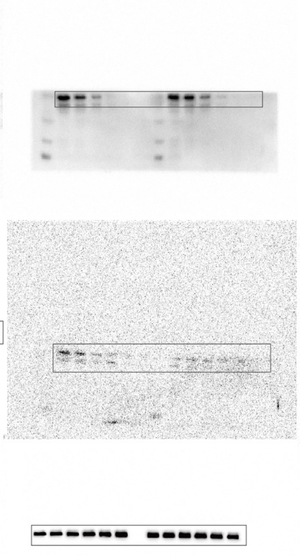

Figure 5D

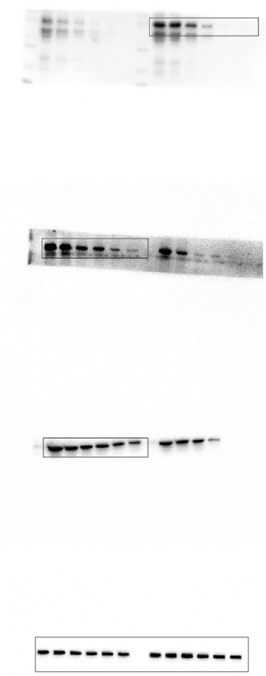

Figure 5E

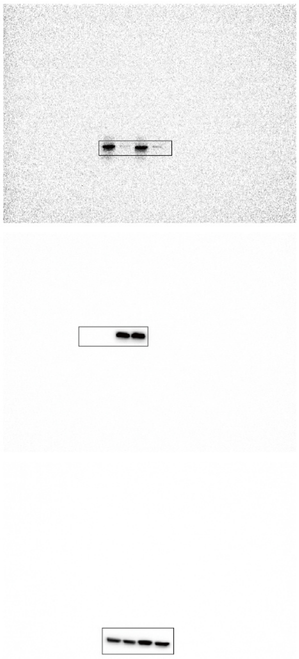

Figure 5E

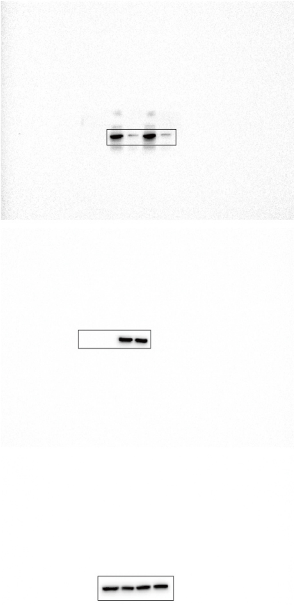

Figure 5F

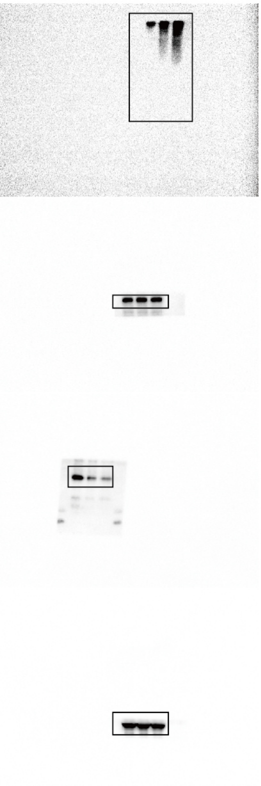

Figure 5G

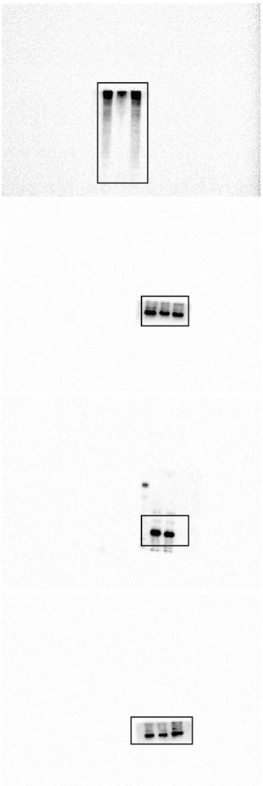

Figure 5H

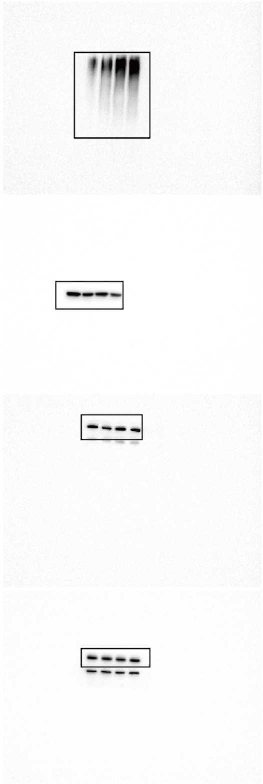

Figure 5I

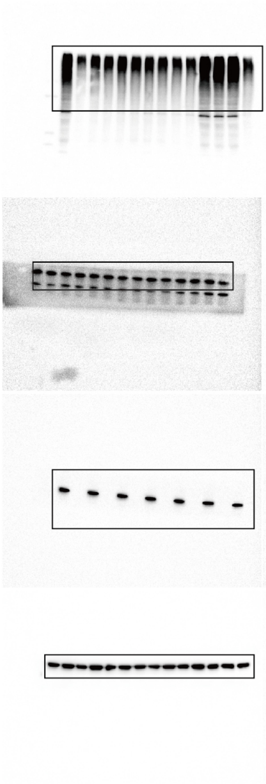

Figure 5I

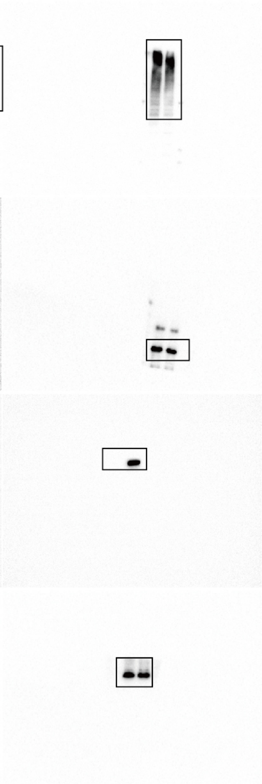

Figure 5J

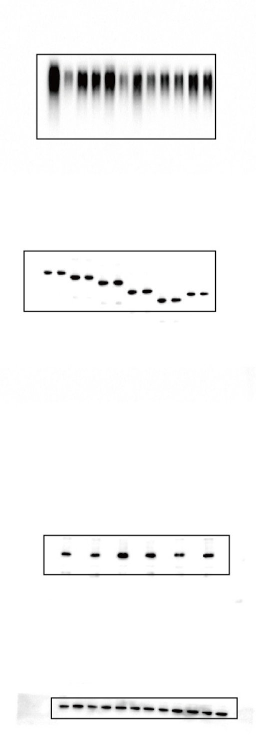

Figure 5L

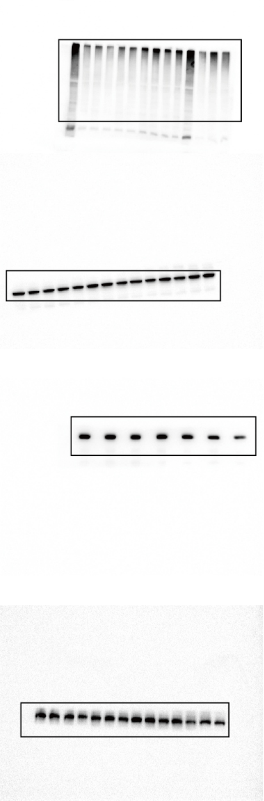

Figure 5L

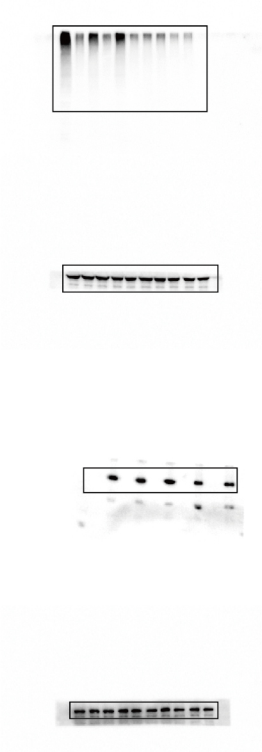

Figure 6F

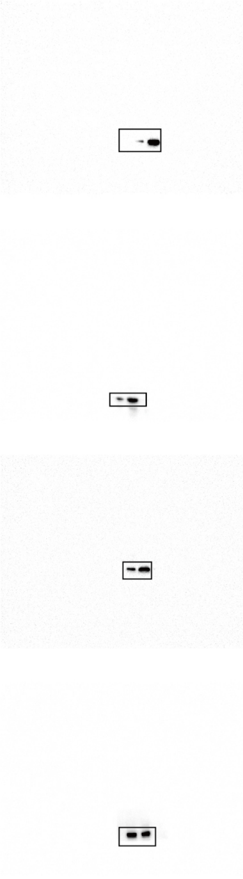

Figure 6G

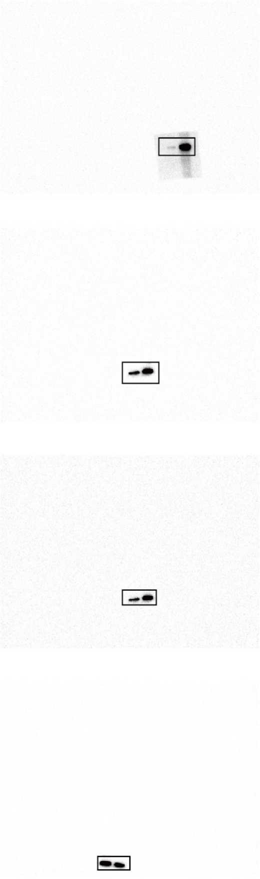

Figure 6I

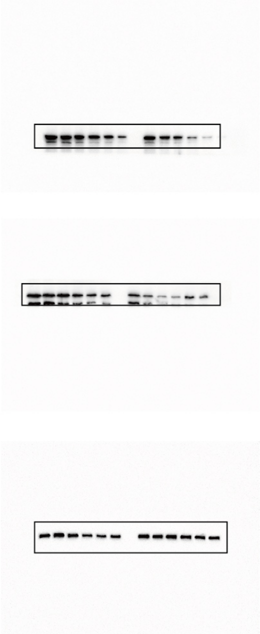

Figure 6I

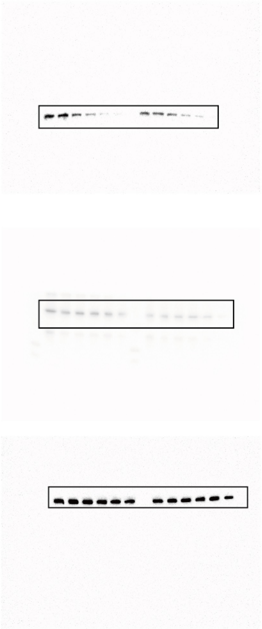

Figure 6H

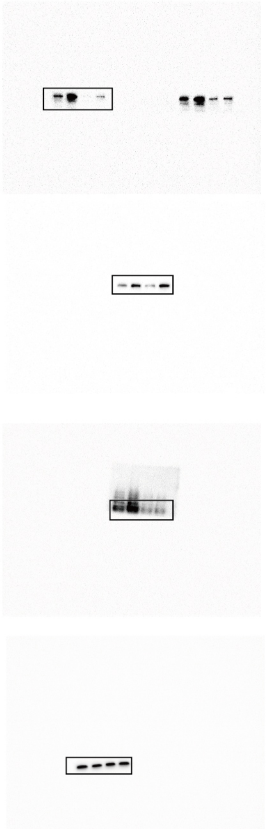

Figure 6J

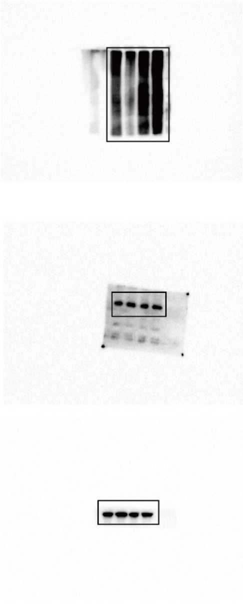

Supplement: Supplementary file 2 — Western blot [file 41420_2025_2869_MOESM2_ESM.pdf]
